# Subtyping psychotic disorders using a data-driven approach reveals divergent cortical and cellular signatures

**DOI:** 10.1038/s41380-026-03774-2

**Published:** 2026-07-23

**Authors:** Lauren D. Hill, Xi-Han Zhang, Baxter P. Rogers, Anna S. Huang, Victoria Fox, Brandee Feola, Stephan Heckers, Avram J. Holmes, Neil D. Woodward

**Affiliations:** 1 Department of Psychiatry and Behavioral Sciences, Vanderbilt University Medical Center, Nashville, TN USA; 2 Department of Psychology, Yale University, New Haven, CT USA; 3 Vanderbilt University Institute of Imaging Sciences, Vanderbilt University Medical Center, Nashville, TN USA; 4 Department of Psychiatry, Brain Health Institute, Rutgers University, Piscataway, NJ USA

**Keywords:** Psychiatric disorders, Neuroscience

## Abstract

Structural brain abnormalities in psychosis are well-replicated but heterogenous posing a barrier to uncovering the pathophysiology, etiology, and treatment of psychosis. To parse neurostructural heterogeneity and assess for the presence of anatomically-derived subtypes, we applied a data-driven method, similarity network fusion (SNF), to structural neuroimaging data in a broad cohort of individuals with psychosis (schizophrenia spectrum disorders (SSD) n = 280; bipolar disorder with psychotic features (BD) n = 101). SNF identified two transdiagnostic subtypes in psychosis (subtype 1: n = 158 SSD, n = 75 BD; subtype 2: n = 122 SSD, n = 26 BD) that exhibited divergent patterns of abnormal cortical surface area and subcortical volumes. Compared to controls (n = 243), subtype 1 showed moderate enlargement of surface area in frontal and parietal areas and larger dorsal striatal volumes, whereas subtype 2 demonstrated markedly smaller surface areas in frontal and temporal areas and subcortical volumes, including hippocampus, amygdala, thalamus and ventral striatum. When comparing subtypes on clinical characteristics, subtype 2 demonstrated more severe negative symptoms, greater neuropsychological impairment, and lower estimated premorbid intellectual functioning. Integrating cell-type data imputed from gene expression in the Allen Human Brain Atlas revealed an association between interregional glial cell and parvalbumin-expressing interneuron abundance and surface area expansion in subtype 1, yet cortical thinning in subtype 2. The largest deviations from controls were present in subtype 2 surface area that spatially coupled with layer 5 glutamatergic neuron abundance, critical for corticostriatal connectivity. These outcomes indicate early diverging neurodevelopmental trajectories of psychosis subtypes, as evidenced by opposing patterns in surface area, cellular fingerprints, and subcortical volumes.

## Introduction

Psychosis disrupts how an individual thinks, feels, and perceives the world. Approximately 1.5 to 3.5% of the population suffers from a psychotic disorder, with schizophrenia spectrum disorders (SSD) and bipolar disorder with psychotic features (BD) being the most severe [1]. Neuroimaging has revealed numerous brain abnormalities in people with psychosis providing critical insights into the pathophysiology, etiology, and treatment of these disorders [2–4]. Alterations in brain structure are among the most prominent findings and include lower cortical thickness in frontal and temporal areas; and smaller subcortical volumes, especially the hippocampus and thalamus [5]. Abnormalities in brain structure are well-replicated but heterogeneous, as demonstrated by three observations. First, the effect sizes are relatively small, ranging from approximately 0.20 to 0.40 standard deviations below normal, indicating significant overlap with healthy individuals [6, 7]. Second, regional brain volumes are more variable in psychotic disorders compared to healthy individuals [8, 9]. Third, recent normative modelling studies have revealed that while the majority of patients have at least one brain region that falls outside the normal range, only approximately 30% of patients demonstrate abnormal deviations for any given brain structure [10, 11]. Combined, these findings strongly suggest the presence of psychosis subtypes characterized by different patterns of brain abnormalities and may provide clues for distinct illness mechanisms.

Two common approaches to dissecting neurostructural heterogeneity in psychosis are to examine the effect of diagnosis, usually by comparing SSD and BD, and illness stage (i.e. early vs. chronic). As SSD and psychotic BD have both shared and distinct clinical symptoms, it is expected that biological similarities and differences can be found. Broadly, structural brain abnormalities are often quantitatively larger in SSD, but qualitatively similar across disorders. For example, both are associated with fronto-temporo-occipital grey matter deficits, however, in SSD, these abnormalities are more severe in a number of regions (e.g., insula) [12, 13]. The modest accuracy of machine learning methods at discriminating between SSD and BD based on brain features underscores the similarities between disorders [14]. A similar pattern emerges when comparing illness stages. While, the magnitude of the abnormalities is often larger in chronic patients, there is considerable overlap between early and chronic stages [15–17]. Consequently, diagnosis and illness stage appear to account for only a modest amount of variability in brain structure abnormalities in psychosis.

To make progress, investigators are using data-driven approaches to parsing heterogeneity. Studies have examined brain structure in subgroups defined based on relatively low dimensional clinical, cognitive, and neurophysiological data [18–20]. The Bipolar and Schizophrenia Network for Intermediate Phenotypes (BSNIP) consortium, which identified three ‘biotypes’ in a large sample of SSD and BD based on a combination of cognitive and neurophysiological biomarkers, is perhaps the most prominent example. Biotypes 1 and 2, which demonstrated more severe impairments on neurophysiology and cognition biomarkers, exhibited widespread reductions in grey matter volume (GMV). In contrast, biotype 3, which demonstrated near normal/normal performance on biomarkers of neurophysiology and cognition, exhibited relatively small alterations in GMV [20]. Subtyping on brain structure exclusively in SSD from the BSNIP consortium revealed moderately overlapping subgroups with BSNIP biotypes, where subgroup 1 displayed widespread reductions in cortical thickness and area and subcortical volumes, while subgroup 2 displayed focal reductions in hippocampal volume and subgroup 3 displayed near normal brain structure [21]. Few studies have applied data-driven approaches to high-dimensional structural neuroimaging data; however, there is growing evidence for at least two SSD subtypes [8, 21–24]. For instance, using HYDRA, a method applied to voxel-wise structural images, Chand et al. [22] identified two subgroups in a cohort of SSD patients; one characterized by widespread lower GMV across cortical and subcortical areas and a second defined by larger basal ganglia volumes in the context of otherwise mostly normal brain structure. These two subgroups were replicated by Chew et al. [25] in deficit and non-deficit SSD and Chand et al. [23] in an independent sample of SSD and linked expression of the subtype associated with widespread smaller GMV to lower cognitive functioning.

Existing studies support the presence of neurostructural subtypes but also have limitations. None included other psychotic disorders such as BD. As noted above, similarities in structural brain abnormalities observed in SSD and BD, along with overlapping symptoms and cognitive deficits, potentially suggests transdiagnostic subtypes. Again, qualitative similarities between early and chronic samples raises the possibility that neurostructural subtypes may transcend illness stage. Indeed, one prior data-driven study found similar neurostructural subtypes for first-episode and midcourse schizophrenia patients [21]. Another limitation is that existing literature focused on cortical volume or thickness exclusively. In the case of the cortex, volume represents the product of two dimensions, surface area and thickness. Cortical thickness and surface area have different genetic architectures, exhibit distinct developmental trajectories, and are differentially related to clinically-relevant variables including cognition and educational attainment [26–28]. Regional surface area exhibits higher heritability compared to thickness and is associated with genes critical to early brain growth and development, including those regulating progenitor cell types in fetal development [29, 30]. Conversely, regional thickness is associated with genes implicated in cell differentiation, migration, adhesion, and myelination, and may reflect environmental influences [29]. Consequently, focusing solely on volume may conflate contributions of surface area and thickness, missing the basic structural elements of the cortex that could help inform disease trajectories and mechanisms.

The current study sought to replicate and extend prior studies in several ways. First, we included a relatively large cohort of individuals with psychosis spanning early and chronic stages of SSD and BD to determine if there are subtypes that transcend diagnostic boundaries and illness stage thereby increasing generalization to the broader psychosis population. Second, we used a relatively new data-driven method, similarity network fusion (SNF) [31], to identify subtypes of psychosis based on multiple brain morphology features, including thickness, surface area, and subcortical volumes. SNF is designed to capture both common and complementary information from different data types providing insight into how informative each feature is to the observed subtypes. Third, the rich phenotypic data available on our psychosis cohort allows us to characterize subtypes on clinical symptoms, neuropsychological functioning, and illness antecedents. Finally, we leveraged microarray data from the Allen Human Brain Atlas (AHBA) and single-nucleus RNA-sequencing data to examine the cell-type correlates of large-scale morphometric pathology seen in psychosis subtypes and bridge gaps across levels of biological systems.

We hypothesized that we would replicate prior studies by identifying two or more anatomically-derived subtypes with at least one demonstrating relatively large and widespread abnormalities in brain structure. We further sought to extend prior data-driven subtyping studies by: 1) establishing which brain features, cortical thickness, surface area, subcortical volumes, or a combination, distinguish subgroups; 2) determining the extent to which anatomically-derived subtypes align with diagnosis and illness stage; 3) clarifying the clinical phenotypes associated with brain-based subtypes; and 4) establishing whether interregional neurostructural profiles of psychosis subtypes spatially relate to cell-type abundances across the brain.

## Methods

### Participants and study procedures

Data was drawn from a repository containing 756 individuals that participated in one of three neuroimaging studies (CT00762866; R01MH070560; R01MH102266) conducted in the Department of Psychiatry and Behavioral Sciences at Vanderbilt University Medical Center (VUMC). All study procedures were approved by the Vanderbilt Institutional Review Board (IRB #s: 080606; 140733). Each participant provided written informed consent to participate in the separate studies and contribute their data to the data repository. See supplemental material for inclusion and exclusion criteria. Briefly, participants with psychotic disorders and healthy individuals were recruited from the Psychotic Disorders Program at VUMC, and surrounding Nashville area via advertisement, respectively. The psychosis cohort included individuals in both early or chronic stages defined as illness duration ≤ 2 or >2 years, respectively. From the initial cohort of 756, 61 were excluded for data quality reasons and 71 for diagnosis not of interest or ineligible (Figure S1), leaving a final sample of 243 healthy individuals and 381 people with a psychotic disorder (SSD n = 280, BD n = 101) (Table 1, Table S1).

**Table 1:** Sample demographics and clinical characteristics.

	Healthy Individuals (n = 243)	Psychotic Disorder (n = 381)	Statistics		
	No. (%)	No. (%)	χ^2^	df	*p*-value
**Sex** (female)	91 (37.45)	137 (35.96)	0.09	1	0.77
**Race**			5.11	4	0.27
White	169 (69.55)	252 (66.14)			
Black	54 (22.22)	104 (27.30)			
Asian	11 (4.53)	8 (2.10)			
American Indian or Alaskan Native	3 (1.23)	4 (1.05)			
Other	6 (2.47)	13 (3.41)			
**Ethnicity** (Hispanic)	12 (4.94)	17 (4.46)	5.22	2	0.07
**Handedness** (right)	223 (91.77)	342 (89.76)	0.36	1	0.55
**Diagnosis** (SSD/BD)	—	280/101			
**Illness-Stage** (early/chronic)	—	236/144			

	M ± SD	M ± SD	*t*	df	*p*-value
**Age** (years)	28.03 ± 9.70	28.36 ± 10.76	−0.39	622	0.69
**Education** (years)	15.36 ± 2.11	13.55 ± 2.18	9.96	590	*<0.001*
**Parental Education** (years)	14.64 ± 2.29	14.70 ± 2.75	−0.25	567	0.80
**Estimated Premorbid IQ**	108.50 ± 7.57	101.99 ± 10.89	8.00	596	*<0.001*
**SCIP** (*z*-score)	0.14 ± 0.63	−0.92 ± 0.93	15.55	610	*<0.001*
**Age of Illness Onset** (years)	—	21.71 ± 6.36	—	—	—
**Duration of Illness** (months)	—	75.13 ± 117.02	—	—	—
**PANSS Positive Symptoms**	—	17.31 ± 8.30	—	—	—
**PANSS Negative Symptoms**	—	14.61 ± 6.82	—	—	—
**PANSS General Symptoms**	—	30.61 ± 8.96	—	—	—
**Premorbid Adjustment** (total)	—	1.37 ± 0.68	—	—	—
**CPZ Equivalents**	—	379.80 ± 494.64	—	—	—

Italics indicate significant *p*-values. Estimated premorbid IQ measured by the WTAR, Wechsler Test of Adult Reading predicted score. Duration of illness was defined as the time at which an individual first met criteria for psychosis (based on extensive interview, review of medical records, and collateral reports) until the date of study enrollment. CPZ, chlorpromazine equivalent. Premorbid adjustment scale available for 217 individuals with psychosis.

*SSD*, Schizophrenia Spectrum Disorder; *BD*, Bipolar with Psychotic Features; *SCIP*, Screen for Cognitive Impairment in Psychiatry; *PANSS*, Positive and Negative Syndrome Scale.

#### Diagnostic and clinical assessments

Diagnoses were confirmed, and ruled out in the case of healthy individuals, by clinical interview with the Structured Clinical Interview for DSM (IV/V) Disorders (SCID) conducted by trained study personnel. Symptoms of psychosis were quantified in the psychosis cohort using the Positive and Negative Syndrome Scale (PANSS) [32]. The Screen for Cognitive Impairment in Psychiatry (SCIP; Purdon, 2005) was administered to all participants to quantify current neuropsychological functioning including five sub-tests assessing immediate and delayed verbal learning, verbal fluency, working memory, and processing speed. Subtest raw scores were *z*-scored based on published norms [33] and averaged to create a SCIP composite score. Premorbid intellectual and social functioning were assessed using the Wechsler Test of Adult Reading (WTAR) [34] and Premorbid Adjustment Scale (PAS), respectively [35].

#### MRI data acquisition, quality assurance, and processing

A high resolution T1-weighted anatomical scan (voxel size=1mm^3^ isotropic) collected in the axial plane was obtained on every participant on one of two identical 3T Philips MRI scanners equipped with a 32-channel head coil located at Vanderbilt Institute for Imaging Sciences (VUIIS) (detailed in supplement). Imaging data were stored and processed on the VUIIS Center for Computational Imaging XNAT [36, 37]. T1-weighted images were visually inspected for neuroimaging artifacts. A nuisance variable (‘scan type’) was included as a covariate in all analyses to account for minor differences in T1 acquisition parameters between studies. The entropy-focus criterion (EFC), a quantitative measure of ghosting and blurring induced by head motion [38], was calculated for each T1-weighted image using MRI Quality Control (MRIQC) [39]. Cortical thickness (CT) and surface area (SA) values and subcortical volumes (SV) were derived from T1-weighted scans using the Desikan-Killiany (DK) atlas in FreeSurfer (v7.2.0) [40, 41]. FreeSurfer segmentations were visually inspected, and 26 subjects were excluded for segmentation failures.

### Data integration and clustering

#### Similarity network fusion

*SNFtool* package v.2.3.1 running in R (version 4.4.1) was used to integrate data types (i.e. CT [68 features], SA [68 features], SV [14 features]) and identify subgroups within the psychosis cohort. Prior to SNF, covariates including age, biological sex, scan type, and EFC from MRIQC [39] were regressed out using general linear models for each brain feature. Residuals were used as input for the SNF analysis. SNF has been previously described [31] (http://compbio.cs.toronto.edu/SNF/SNF/Software.html) and detailed in the supplement. Briefly, SNF first creates separate similarity networks for each data type and then integrates these networks using a nonlinear combination method to iteratively fuse each data type network into a single, global network, representing the full spectrum of included features. Across iterations, low weight edges (i.e., similarities between participants) are removed to reduce noise, while high weight edges are added together. Similarity matrices for each data type were calculated using Euclidean distance with a nearest neighbors values of 30 (K) and a normalization parameter of 0.6 (alpha), based on recommended ranges in the *SNF tool* R package, 10–30 for K (sample size/10) and 0.3–0.8 for alpha [31]. Cluster number was chosen using the *estimateNumberOfClustersGivenGraph* function to calculate eigen-gaps and rotation costs for each K-alpha combination. We tried 126 combinations of K and alpha hyperparameters (K = 10–30; alpha=0.3–0.8), consistent with prior applications [42] to determine cluster number stability. Normalized mutual information (NMI) was used to determine the relative importance of each brain feature for determining clustering. Higher NMI scores indicate greater contribution to participant similarity (*rankfeaturesbyNMI* function). Spectral clustering (*spectralClustering* function) was then applied to identify subgroups based on participant similarity matrices determined based on all 150 features across 1000 iterations of resampling 80% of participants. A silhouette plot, derived from the clustering consensus matrix, quantified similarities between participants within a given subgroup compared to participants in all other subgroups.

#### Cluster stability

SNF cluster stability was assessed via resampling-based stability testing with 1000 iterations. For each iteration, 80% of participants were randomly sampled, their brain morphology features integrated using SNF, and clustered into subgroups. Three metrics of cluster stability were calculated for each iteration, 1) the percentage of time each participant clustered with every other participant (when pairs were included in the same resampling iteration); 2) the Adjusted Rand Index [43, 44] which measures the overlap between clusters to determine stability of the data-driven model (range: 0 to 1, 0=completely random overlap, 1=identical clustering across iterations); and 3) the percentage with which each model feature remained among the top 35 features across iterations based on NMI scores.

### Statistical analyses

Continuously distributed and dichotomous variables were analyzed using independent groups *t*-tests/general linear regression and Chi-square analyses, respectively. Age, biological sex, scan type, scan quality (i.e., EFC), and, in the case of subcortical volumes, estimated total intracranial volume (ICV), were included as covariates in the analysis of FreeSurfer derived brain structure dependent variables. Multiple comparisons were controlled for using the false discovery rate (FDR) (*p*_FDR_ < 0.05) [45]. Cohen’s *d* effect sizes for each region of interest were computed based on the group contrast *t*-statistics [46].

### Cell-type imputation and analysis

Cell-type density maps were downloaded from Zhang et al. (see Extended Data Fig. 1A) [47]. Briefly, gene expression signatures of 24 neuronal and nonneuronal cell-types were derived from single-nucleus RNA-sequencing data sampled from eight cortical areas (detailed in supplement). Signatures were then used to impute cell-type fractions within each of the cortical 1676 bulk tissue samples. Each bulk tissue sample was registered to MNI space and microarray-sequenced for the total gene expression, from which the linear combination of the 24 cell-types was deconvolved based on their gene expression signatures (detailed in supplement). The 24 cell-type fractions of each microarray sample were registered to the closest DK parcel based on the vertex ID of the sample. Parcel-level cell-type fractions were obtained by taking the average of samples registered to the same parcel.

Following protocols detailed in Zhang et al. [47], cell-types’ combinational correlation to interregional neurostructural deviations was estimated via permutational canonical correlation analysis (PermCCA), which examines the linear combination of cell-type interregional abundances that maximally correlate with each subtype neurostructural profile (CT and SA). Statistical significance of the canonical variates was tested via a permutation method that controls for cortical spatial autocorrelation, where the null distributions of cell-type fractions were generated from the Baum method [48] (Baum method projects the parceled cell fractions to the surface mesh, which was subsequently permuted while retaining the spatial adjacency in a spinning manner, then re-parcellated by taking the modal value of the vertices in each parcel). FDR was applied to correct for four comparisons. The cell-types’ linear combinational correlation to each neurostructural profile was measured by the cell-type composite score. Each cell-type’s contribution to this correlation was measured by loadings, the correlation between cell-type abundance distribution and neurostructural profile canonical variate.

## Results

### Brain structure in psychosis

**Fig. 1:**
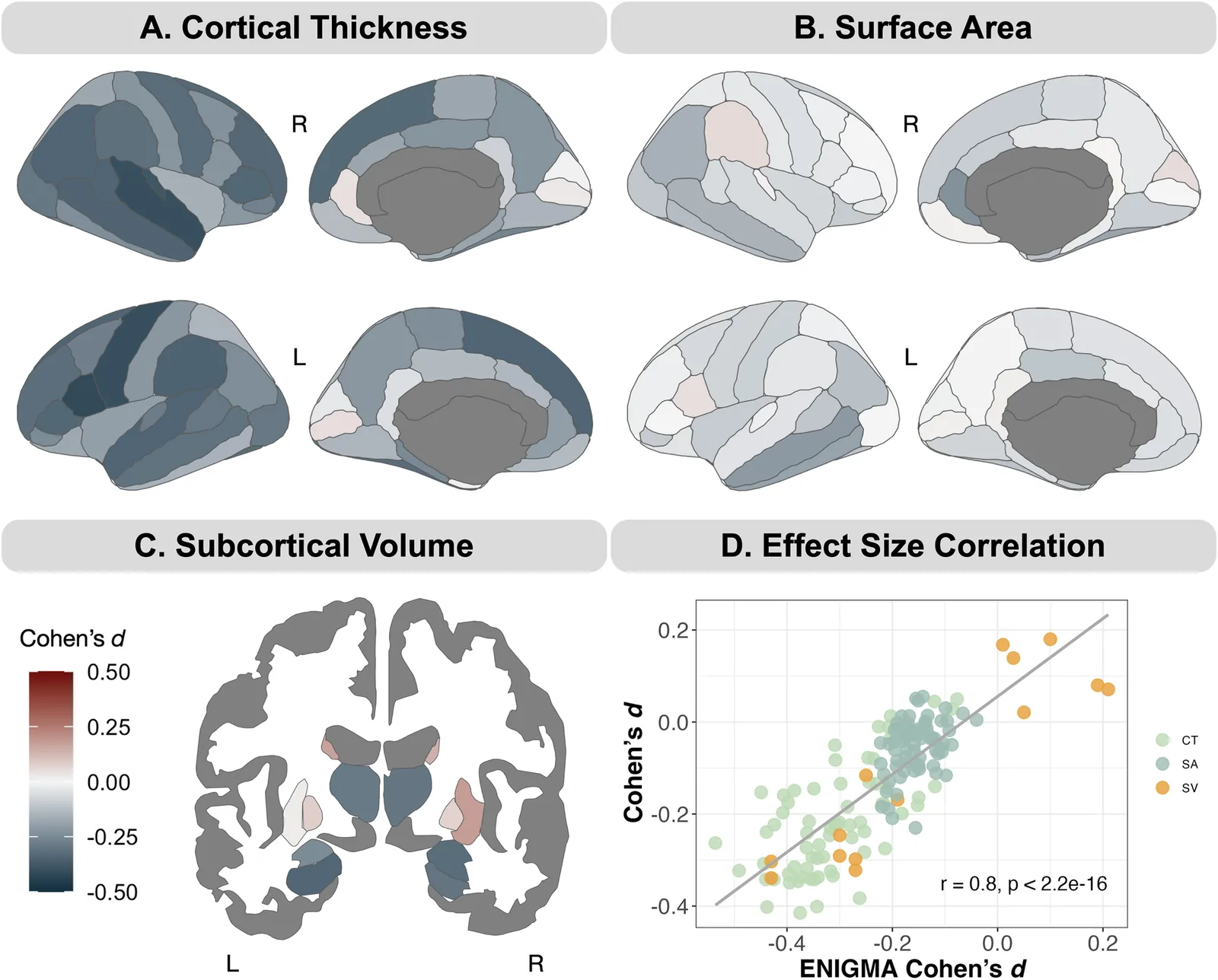
Maps of regional Cohen’s *d* effect sizes for psychosis vs. healthy individual contrast. **A**-**C** Regions with lower and higher cortical thickness, cortical surface area, and subcortical volume in psychosis are depicted in cool and warm colors, respectively. **D** Pearson’s correlation between regional effect sizes in the current study and ENIGMA Schizophrenia.

To establish whether structural abnormalities in our psychosis cohort are representative of the broader psychosis population, we calculated effect sizes (ES) for the psychosis vs. healthy individuals’ contrast for all brain morphology features and compared them to the ENIGMA Schizophrenia meta-analyses [6, 7]. In Fig. 1A, psychosis was associated with widespread cortical thinning, smaller surface areas, and smaller subcortical volumes. Consistent with ENIGMA Schizophrenia, ESs were in the small to medium range (i.e., 0.20 to 0.40). ESs in the current sample correlated highly with ENIGMA Schizophrenia meta-analyses (*r* = 0.80, *p* < 2.2^−16^, Fig. 1D) indicating that the structural brain abnormalities observed in our cohort was very consistent with the general pattern observed in psychosis.

### Composition and stability of neuroanatomically-derived clusters in psychosis

**Fig. 2:**
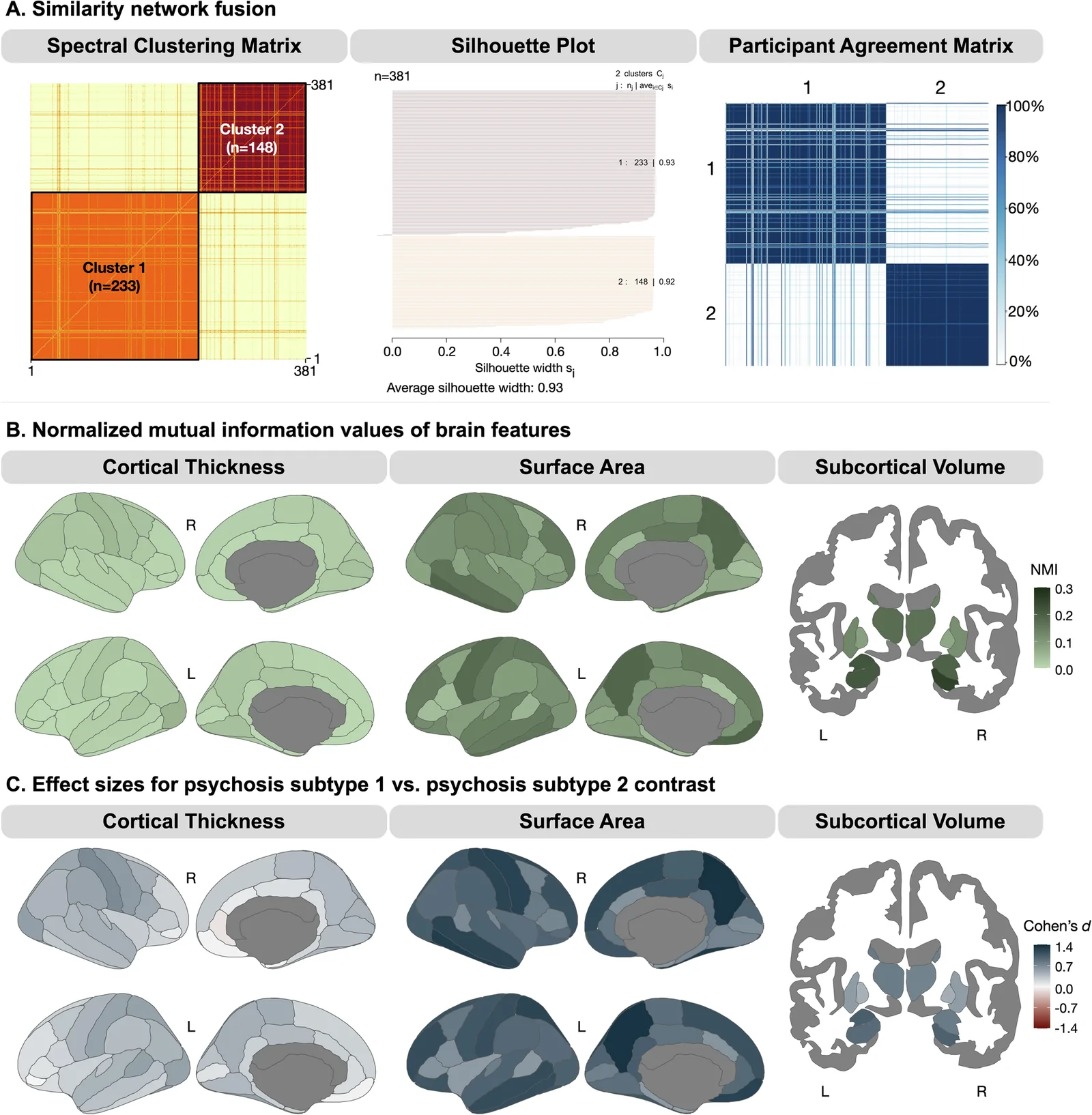
Data-driven similarity network fusion analysis of brain morphology features revealed two clusters in psychosis. **A** Participant-to-participant SNF-combined similarity matrix after clustering into two subtypes of psychosis individuals. Silhouette plot illustrating the similarities between participants within a cluster versus the other cluster. Cluster number: n of cluster | average cluster silhouette width. Participant agreement matrix, how often each participant clustered with one another across resampling 80% of psychosis individuals 1000x (dark blue = higher percentage of instances that each pair of participants clustered together. **B** Normalized mutual information (NMI) values across brain regions. Surface area (SA) and subcortical volume (SV) had higher NMI values compared to cortical thickness (CT), indicating greater importance in determining data-driven subtypes. **C** The largest Cohen’s *d* effect sizes for subtype 1 vs. subtype 2 were found in SA (*d* = 0.52 to 1.36) and SV (*d* = 0.43 to 1.01) compared to CT (*d* = 0.02 to 0.69). Cool colors = greater reductions in CT, SA, and SV in subtype 2 vs. subtype 1, uncorrected for multiple comparisons.

SNF and spectral clustering revealed two participant similarity network clusters (Fig. 2A) comprised of n = 233 and n = 148 individuals (hereafter referred to as subtypes 1 and 2, respectively) based on eigen-gaps and rotation costs (see supplemental material for SNF results in SSD only). The average silhouette width was 0.93 indicating a strong cluster structure (Fig. 2A, silhouette plot).

Resampling-based stability testing with 1000 iterations, each using a random sampling of 80% of participants, revealed that clusters were highly stable. On average, 92% of the time any given participant clustered with each other participant within their data-driven group, while only 8% of the time an individual was clustered with a participant in another group (Fig. 2A, participant agreement matrix). The Adjusted Rand Index was 0.85 across 1000 iterations, indicating a high level of agreement between clustering solutions considering chance agreement.

### Relative contribution of brain features in defining neuroanatomical subtypes of psychosis

In Fig. 2B, surface areas and subcortical volumes had higher NMI scores, indicating higher relatively importance in determining clusters than thickness (Table S6 for all feature NMI values). The top 10 model features, based on NMI scores, included subcortical volumes (hippocampus, amygdala, thalamus) and surface area of the medial orbitofrontal, inferior temporal, and precuneus. No thickness features appeared in the top 10 or when the range was extended to the top 35 (Table S7). Resampling-based stability testing indicated that the top-ranking features based on NMI scores were consistent across iterations. On average, the top 10 features remained among the top 35 more than 99% of the time across permutations (Figure S2).

ESs for the difference between subtypes were calculated for all brain features to further illustrate the relative importance of regional brain features that differentiate the subtypes. In Fig. 2C, and consistent with the NMI scores, ESs were very large (ES ~ 1–1.5) for surface area, especially frontal, temporal, and midline areas, and subcortical volumes, whereas ESs were considerably smaller for thickness.

### Neurostructural profiles of data-driven subtypes

**Fig. 3:**
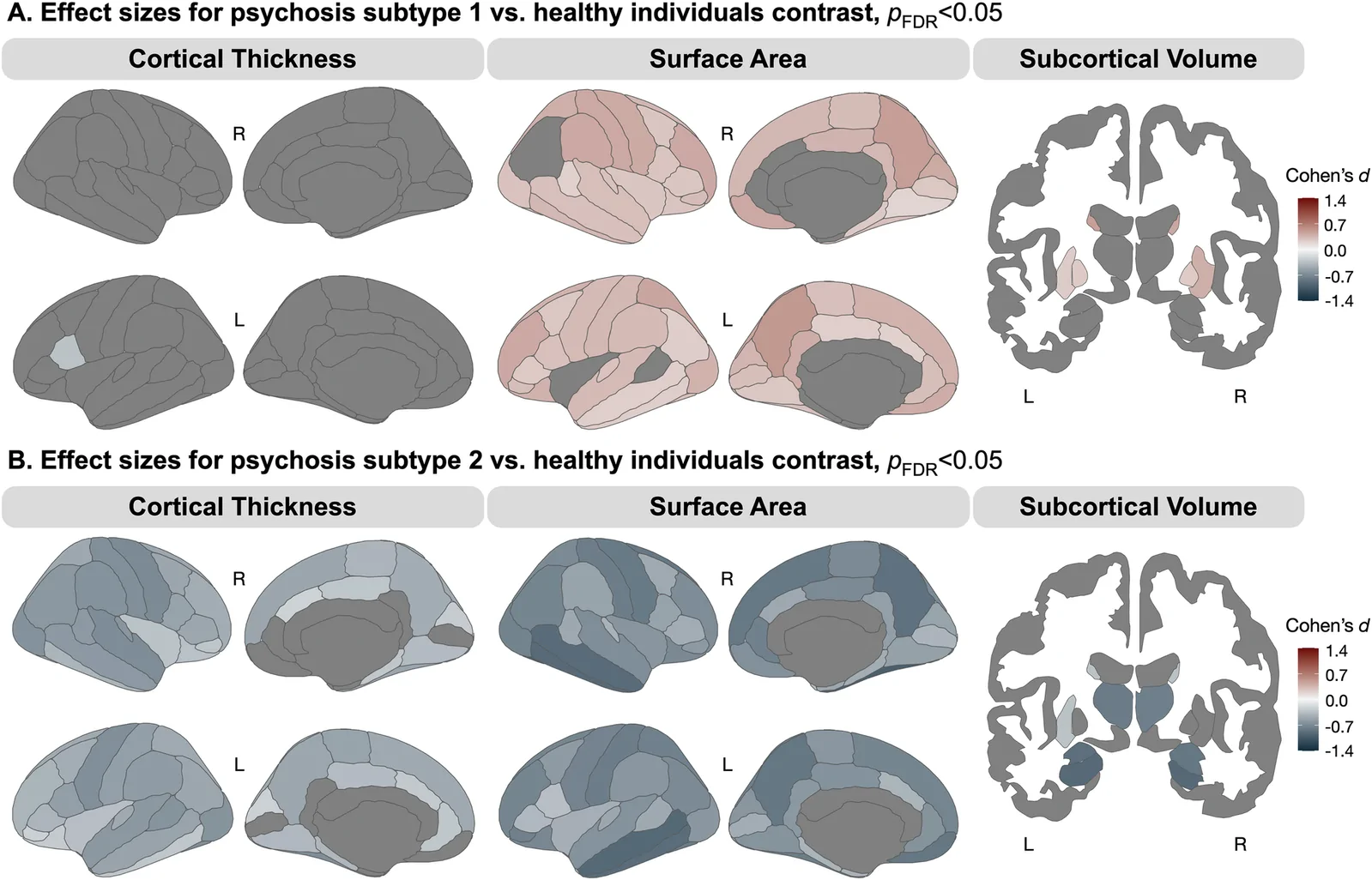
Data-driven psychosis subtypes demonstrate distinct neurostructural profiles compared to healthy individuals. **A** Compared to healthy individuals, psychosis subtype 1 demonstrated small to moderate (*d* = 0.16 to 0.52), but significantly larger surface area across the cortex and larger basal ganglia volumes. **B** Compared to healthy individuals, psychosis subtype 2 demonstrated widespread lower surface area and cortical thickness, and smaller subcortical volumes. Effect sizes were larger for surface area and subcortical volume (*d* = −0.33 to −1.00) versus thickness (*d* = −0.23 to −0.69).

Subtype neurostructural profiles were established by comparing each subtype to healthy individuals. In Fig. 3, subtype 1 demonstrated small to medium (ES = 0.16 to 0.52) enlargement of surface areas in most cortical areas, lower thickness in a single region of the prefrontal cortex, and larger basal ganglia volumes (caudate, putamen, globus pallidum). In contrast, subtype 2 was characterized by widespread smaller surface area and lower thickness, and smaller subcortical volumes. ESs were notably larger for surface areas and subcortical volumes (ES = −0.33 to −1.00) relative to thickness (ES = −0.23 to −0.69). For complete statistical results, including unthresholded statistical maps and SSD-only subtype profiles, see supplemental material.

### Clinical characteristics of neuroanatomically-derived psychosis subtypes

**Fig. 4:**
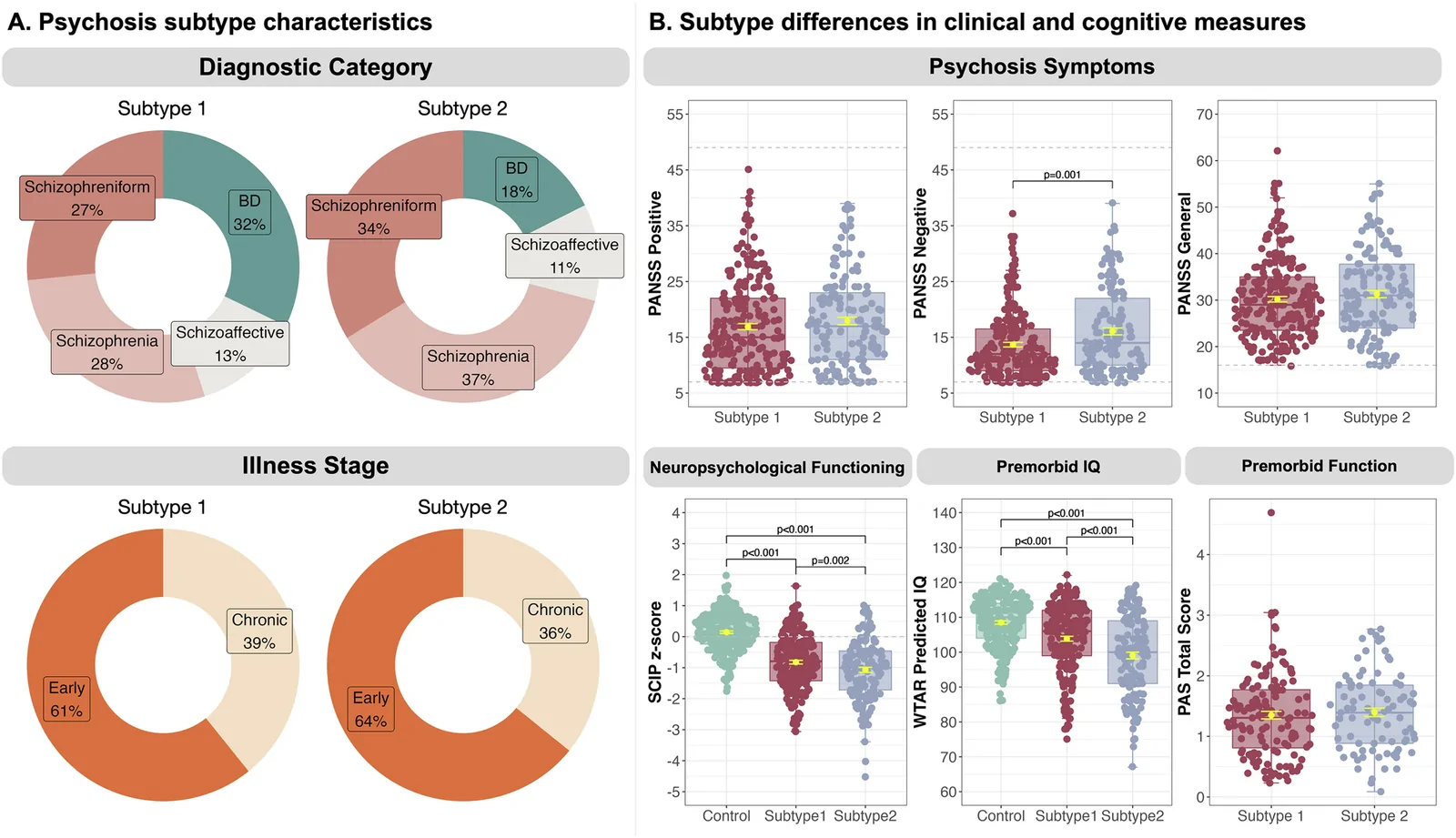
Clinical characteristics of neuroanatomically-derived psychosis subtypes. **A** Psychosis subtypes displayed similar proportions of psychotic disorder diagnoses and illness stages. **B** Subtype 2 displayed higher PANSS negative symptom scores compared to subtype 1, more severe cognitive impairment and lower premorbid intellectual functioning compared to both subtype 1 and healthy individuals.

Subtype clinical characteristics and demographics are presented in Table S5. Proportions of psychotic disorder illness stage were similar across subtypes, while proportions of SSD and BD diagnosis were different (χ^2^(1, *N* = 381) = 9.93, *p* = 0.002) (Fig. 4A). There was no difference between subtypes in duration of illness and average dosage of antipsychotic medication (in CPZ equivalents).

Significant differences were observed between subtypes in severity of clinical symptoms and cognitive impairment (Fig. 4B, supplemental material). Subtype 2 exhibited higher PANSS negative symptom scores versus subtype 1 (*b* = 2.3, SE = 0.7, *p* = 0.001), and more severe cognitive impairment and lower premorbid intellectual functioning versus both subtype 1 (*b* = −0.26, SE = 0.08, *p* = 0.002 and *b* = −4.91, SE = 1.02, *p* < 0.001, respectively) and controls (*b* = −1.22, SE = 0.08, *p* < 0.001 and *b* = −9.46, SE = 1.01, *p* < 0.001). PAS scores did not differ between subtypes.

### Multivariate cellular profiles of psychosis subtypes

**Fig. 5:**
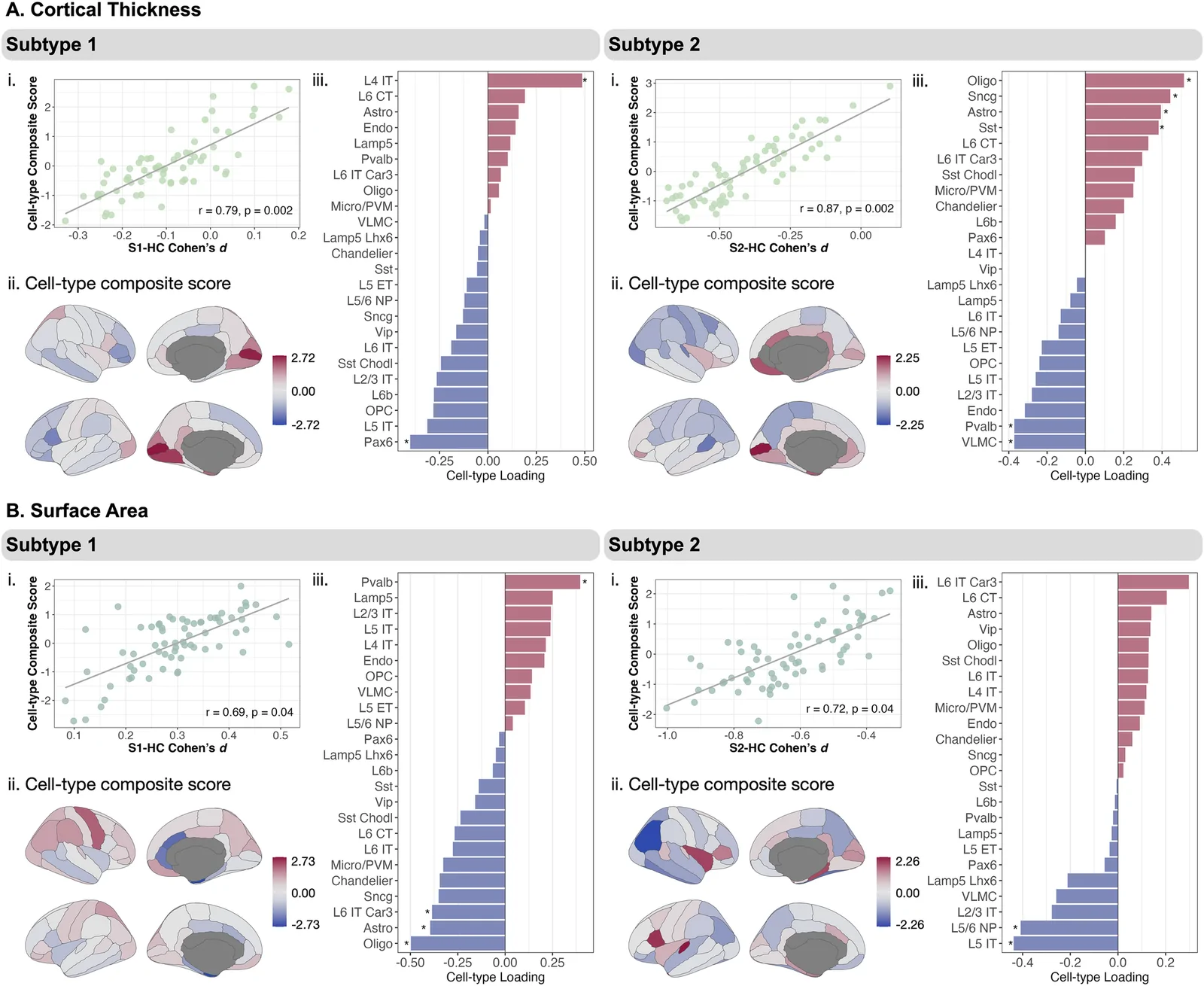
Multivariate cellular profiles follow spatial topography of neurostructural abnormalities in psychosis subtypes. Psychosis subtypes displayed differential spatial relationships between multiple cell-types and cortical thickness (panel **A**) and surface area (panel **B**) effect size maps (Cohen’s *d*, see Fig. 4). **A**, **B** i: scatter plots for each subtype displaying the results of PermCCA, where the effect size map was positively related with a composite score of cell-type abundances (95% CI). **A**, **B** ii: cell-type composite scores associated with each effect size map projected to the cortical surface.  **A**, **B** iii: canonical loadings of each cell-type to the composite score, significant cell-types indicated with an *, red = positive associations and blue = negative associations.

Using PermCCA, we examined interregional associations between subtype neurostructural profiles and multiple cell-type abundances (Fig. 5). Cell-type composite scores were correlated with both thickness and surface area ES contrast maps. Regions in subtype 1 with more severe thinning overlap regions of greater PAX6-expressing inhibitory interneuron but lower layer 4 intratelencephalic-projecting (L4 IT) excitatory neuron abundance (*r* = 0.79, *p*_FDR_ = 0.002). Regions in subtype 2 with more severe thinning overlap regions of greater parvalbumin-expressing (PVALB) inhibitory interneuron and vascular leptomeningeal cell (VLMC), but lower oligodendrocyte (Oligo), SNCG-like (SNCG) inhibitory interneuron, astrocyte (Astro), and somatostatin-expressing (SST) inhibitory interneuron abundance (*r* = 0.87, *p*_FDR_ = 0.002). Regions in subtype 1 with expanded surface area overlap regions where PVALB is abundant, but layer 6 IT (L6 IT Car3) excitatory neuron, Astro, and Oligo are less prevalent (*r* = 0.69, *p*_FDR_ = 0.04). Regions in subtype 2 with smaller surface area overlap regions where layer 5/6 near-projecting (L5/6 NP) and layer 5 IT (L5 IT) excitatory neurons are abundant (*r* = 0.72, *p*_FDR_ = 0.04).

## Discussion

In a broad cohort of individuals with psychosis spanning early and chronic stages of both SSD and BD, we assessed the presence of neuroanatomical subtypes using a relatively new data-driven approach and integrated clinical and cell-type data to provide critical insight into potential diverging etiological pathways. We identified two distinct psychosis subtypes driven by heterogeneity in surface area, notably medial orbitofrontal and precuneus, and bilateral temporal and frontal regions; and subcortical volumes of the hippocampus, amygdala, and thalamus. Subtype 1 exhibited a small, but widespread increase in surface area and larger basal ganglia volumes (vs. controls), whereas subtype 2 exhibited medium to large reductions in surface area and subcortical volumes. These findings align with a recent ENIGMA meta- and mega-analysis showing the greatest multimodal structural brain variability in schizophrenia for frontotemporal and subcortical structures (e.g., hippocampus, putamen) [49]. In contrast to the divergent pattern observed for surface area and subcortical volumes, widespread cortical thinning was observed in both subtypes but was more severe and widespread in subtype 2. Reduced thickness in both subtypes in the context of divergent patterns in surface area likely explains the widespread modest reduction in thickness and variable effects in surface area seen in the overall psychosis cohort versus healthy individuals contrast. Subtypes were highly stable and not a consequence of demographic factors robustly associated with brain structure (e.g., age and sex) or illness chronicity. Subtype 1 had a higher proportion of BD (32% vs. 18% subtype 2), however, the relative proportions of SSD diagnoses were similar across subtypes. Overall, the current findings provide strong support for two anatomically-derived subtypes that transcend illness stage and diagnosis.

Our findings are strikingly similar to prior data-driven approaches to parsing neurostructural heterogeneity in psychosis (see review by Gonul and colleagues [50]). Subtype 1 replicates Chand et al. [22, 23] showing a schizophrenia subtype marked by enlarged basal ganglia volumes and Jiang et al. [8] trajectory-based models showing a subtype marked by enlarged striatum and cortical-predominant loss. A selective increase in striatal volume may be attributed to an excess of dopamine and dopamine receptors in schizophrenia [51, 52]. There is speculation that for a subgroup of patients, this observed increase in D2 receptor binding may be genetically determined [53, 54] and persist in those even without antipsychotic treatment [8]. We show that larger basal ganglia volumes are accompanied by widespread, but modest increase in surface area and cortical thinning that, while significant after correction for only one brain region, was also present throughout the cortex. Increased surface area and decreased thickness potentially explain why prior voxel-wise studies of GMV were unable to detect this pattern, as cortical volume reflects the product of both. Another dissociating aspect of our study is that our psychosis cohort included BD. Surface area abnormalities in SSD [7] and BD [13] are highly variable, with small effect sizes, particularly for BD, which has been linked to both regional increases and decreases [13]. This pattern of widespread thinner cortex and expanded area, notably in the precuneus and supramarginal gyrus, has been shown in non-deficit, but not deficit subgroups of schizophrenia [55]. Subtype 1 may therefore capture a subset of patients aligned with neurostructural patterns previously identified in BD and non-deficit schizophrenia. Subtype 2 (39% of our sample) is consistent with findings showing widespread reductions in cortical volume and some subcortical regions [21–23] that may first appear in the hippocampus [8]. Subtype 2 replicates Xiao et al. [21] showing a schizophrenia subtype marked by widespread lower cortical thickness and surface area, and smaller subcortical volumes, notably the thalamus. We extend these findings to show that reductions are present in both SSD and BD. While a smaller portion of our sample, subtype 2 reflects the more typical case-control findings in SSD, but with much larger ESs (>0.50 vs. 0.20–0.40) suggesting a more impaired subtype marked by prominent reductions in surface area, as well as thickness and subcortical volumes. Consistent with greater neuroanatomical burden, subtype 2 displayed worse negative symptoms, and greater impairments in neuropsychological functioning and premorbid IQ versus subtype 1.

Overlapping deficits in cortical thickness in the context of distinct abnormalities in surface area may indicate both overlapping and unique mechanisms leading to structural abnormalities in each subtype. The synaptic pruning hypothesis posits that normative cortical thinning and remodeling that occurs from adolescence to young adulthood, when psychosis is most likely to emerge, is compromised in SSD [56]. This hypothesis is supported by longitudinal studies showing that the rate of cortical volume loss during this transitional period is higher in individuals at high-risk for psychosis that later transition to a psychotic disorder compared to both high-risk non-converters and healthy people [57]. Potentially the cause of this abnormality, whether it’s overactive synaptic pruning or a different mechanism(s), is shared across subtypes, but is more severe in subtype 2. In contrast, the striking differences in surface areas strongly implies disparate trajectories in early brain development. Surface area increases dramatically from the 3^rd^ trimester until about 24-months postnatally after which it continues to increase, but at a much lower rate until peaking around age 11 [58, 59]. Surface area development is complex and incompletely understood, involving neurogenesis and neuronal migration, including formation of cortical columns and relative proportion of basal radial glial cells which promote tangential spread of migrating neurons and cortical folding during fetal gestation [60–62]. Expanded surface area has been reported among individuals at familial high-risk of schizophrenia [63, 64] and is hypothesized to reflect aberrant processes in cell migration early in development (pre/perinatal) or later normative processes to reduce surface area (i.e., synaptic pruning), however, longitudinal research is needed to better elucidate the role of surface area in the pathological process of psychosis [65]. Postnatal factors such as psychosocial stress, variability in cognitive and linguistic stimulation, and environmental exposures (e.g., lead), some of which are anchored in racial inequalities, may also impact structural brain development [66–68]. Notably, subtype 2 comprised a higher proportion of Black individuals than subtype 1. Our findings suggest one or more of these processes is disrupted in a manner that promotes overgrowth in subtype 1 and undergrowth in subtype 2. Interestingly, both 22q11.2 deletion syndrome, which increases risk for psychosis approximately 30-fold, and copy number variants linked to developmental disorders are associated with smaller surface area, suggesting differences in genetic risk factors between subtypes [69, 70].

To bridge gaps across levels of biological systems, we used transcriptional data from the AHBA to examine how cell-type abundances spatially relate to subtype structural abnormalities. In both subtypes, greater abnormalities (expanded surface area in subtype 1, and greater thinning in subtype 2) were topographically linked with entorhinal/parahippocampus, anterior cingulate, and insula regions that display less glial cell (Oligo and Astro) abundance, and transverse temporal, parietal, and middle frontal regions that display greater PVALB abundance. While we cannot infer specific mechanisms, this finding emphasizes the potential functional relevance of glial cells and PVALB neurons in the divergent patterns of subtype abnormalities. A study examining transcriptome-MRI associations found marked heterogeneity in cell-type specific associations with cortical thickness in schizophrenia and suggests the presence of three cellular subtypes [11]. Here, subtype 2, aligns with a cellular subtype marked by severe and widespread thinning present in brain regions where Astro is less prevalent and inhibitory (e.g., PVALB) neurons are more abundant. Subtype 1, potentially overlaps with a cellular subtype marked by milder thinning, however, given the discrepancy in granularity of our cell-type analysis and Di Biase et al. [11] (24 vs. 7 cell-types), we are unable to discern whether the positive association we found with L4 IT and negative association with Pax6 aligns. Moving beyond the extant literature on thickness, we suggest that glial cells and PVALB, in a subset of individuals with psychosis, may be differentially related to surface area expansion, again pointing to early diverging neurodevelopmental trajectories.

The largest ESs were found in surface area for subtype 2, where smaller area was topographically linked with parietal, superior temporal, and medial/lateral orbitofrontal regions that display greater L5 IT and L5/6 NP abundance. This finding suggests a relationship between layer 5 pyramidal neurons and surface area in the most impaired psychosis subtype. Layer 5 pyramidal neurons are critical for cortical input and output, where feedforward sensory information is integrated at their basal dendritic compartment, and feedback contextual information is integrated at their apical dendrites [71]. L5 IT neurons, located in superficial layer 5 (L5a), project intracortically and to the striatum, and receive primary input from higher-order thalamic nuclei [72]. These corticostriatal loops involving the prefrontal cortex, striatum, and thalamus, have long been hypothesized to be affected in schizophrenia and related to cognitive dysfunction [73, 74]. Spatial distributions of L5 IT neurons have also shown to map onto functional brain networks engaged in goal-oriented cognition (frontoparietal network), and visuospatial attention (dorsal attention), while co-expression of L5 IT and L5/6 IT neurons map onto a network engaged in recollection and mentalizing (default mode) [47]. As such, layer 5 pyramidal neurons may be implicated in a more impaired subtype of psychosis.

Strengths of the current work are the inclusion of a relatively large, diverse psychosis cohort, use of a data-driven technique to parse heterogeneity in multiple brain morphology measures, rich phenotypic data to thoroughly characterize subtypes, and the use of transcriptomics to develop cellular fingerprints of subtype abnormalities. Considering these strengths, several limitations should be acknowledged. The data used was cross-sectional, and future longitudinal studies should examine changes in subtypes over time, and how that relates to clinical phenotypes. Second, while we conducted robust resampling-based stability testing, the identified subtypes should be replicated in new samples to test generalizability. Third, the human microarray gene expression data obtained from bulk samples of six postmortem brains from the AHBA dataset were not from individuals with psychosis. Future studies should examine how subtype abnormalities map onto cell-type distributions in people with psychotic disorders. Fourth, cell-type abundances are imputed from bulk-tissue microarray data that does not provide a direct estimate of gene transcription, but rather within-probe differences across samples. Bulk samples require aggregation into parcels to obtain robust estimates across donors, which limits the spatial resolution. Fifth, prior to SNF data integration, covariates including age and biological sex were regressed out. Future studies should consider clustering analyses in males and females separately within specific age bands. Finally, an important future direction will be to examine additional morphometric brain features related to surface area including cortical folding and developmental scaling of regional and global brain size.

## Supplementary information

Supplemental Material

## Data Availability

Neuroimaging data included in this study are available from the corresponding author upon reasonable request and subject to a data-sharing agreement. Human microarray gene expression data obtained from bulk samples of six postmortem brains are available from the Allen Human Brain Atlas (https://human.brain-map.org). Single-nucleus RNA-sequencing (snRNA-seq) profiles can be obtained from refs. [47, 75].
