# Supplementary material for: Subtyping psychotic disorders using a data-driven approach reveals divergent cortical and cellular signatures
Source: Mol Psychiatry. 2026 Jul 23;31(12):7219–29. doi: 10.1038/s41380-026-03774-2 (PMC13736653; doi:10.1038/s41380-026-03774-2)
Supplement: Supplementary file 1 [file 41380_2026_3774_MOESM1_ESM.docx]

**SUPPLEMENTAL INFORMATION**

**Subtyping psychotic disorders using a data driven approach reveals divergent cortical and cellular signatures**

Lauren D. Hill, Xi-han Zhang, Baxter P. Rogers, Anna S. Huang, Victoria Fox, Brandee Feola, Stephan Heckers, Avram J. Holmes, Neil D. Woodward

**SUPPLEMENTAL CONTENT**

SUPPLEMENTAL TEXT

- Participant information

- MRI data acquisition

- Similarity network fusion

- Cell-type imputation

- Clinical characteristics of psychosis subtypes

SUPPLEMENTAL FIGURES

**Figure S1.** Sample inclusion/exclusion flow chart.

**Figure S2.** Stability of the top 35 brain features contributing to data-driven psychosis subtypes.

**Figure S3.** Unthresholded effect size brain maps of psychosis subtype 1 and 2 vs. healthy controls.

**Figure S4.** Data-driven similarity network fusion analysis of brain morphology features revealed two clusters in SSD individuals.

**Figure S5.** Data-driven SSD subtypes demonstrate notably similar neurostructural profiles (vs. healthy controls) as those identified in the full psychosis sample.

**Figure S6.** Unthresholded effect size brain maps of SSD subtype 1 and 2 vs. healthy controls.

**Figure S7.** Cortical thickness regions of interest, left hemisphere, density plots by psychosis subtype.

**Figure S8.** Cortical thickness regions of interest, right hemisphere, density plots by psychosis subtype.

**Figure S9.** Surface area regions of interest, left hemisphere, density plots by psychosis subtype.

**Figure S10.** Surface area regions of interest, right hemisphere, density plots by psychosis subtype.

**Figure S11.** Subcortical volume regions of interest density plots by psychosis subtype.

SUPPLEMENTAL TABLES

**Table S1.** Sample demographics and clinical characteristics by diagnostic category.

**Table S2.** Cortical thickness comparisons among patients (SSD and BD) and healthy controls.

**Table S3.** Surface area comparisons among patients (SSD and BD) and healthy controls.

**Table S4.** Subcortical volume comparisons among patients and healthy controls.

**Table S5.** Sample demographics and clinical characteristics by data-driven psychosis subtype.

**Table S6.** Normalized mutual information (NMI) values for all in-model brain features.

**Table S7.** Psychosis subtype comparisons on top 35 ranking model features contributing to participant similarity as determined by NMI.

**Table S8a.** Cortical thickness comparisons among data-driven subtype 1 and healthy controls.

**Table S8b.** Cortical thickness comparisons among data-driven subtype 2 and healthy controls.

**Table S9a.** Surface area comparisons among data-driven subtype 1 and healthy controls.

**Table S9b.** Surface area comparisons among data-driven subtype 2 and healthy controls.

**Table S10a.** Subcortical volume comparisons among data-driven subtype 1 and healthy controls.

**Table S10b.** Subcortical volume comparisons among data-driven subtype 2 and healthy controls.

REFERENCES

SUPPLEMENTAL TEXT

**Participant information.** Psychiatric diagnoses were confirmed, and ruled out in the case of healthy individuals, via interview with the Structured Clinical Interview for DSM-IV/V (SCID-IV/V) [1] conducted by trained research staff. Exclusion criteria were nearly identical across the three neuroimaging studies and included age less than 16 or older than 65 (age 55 in the case of R01MH102266); history of significant head trauma, medical illness or central nervous system disorder (e.g., epilepsy); substance abuse within the last one month for schizophrenia participants (three months in the case of R01MH102266) or lifetime history of substance abuse/dependence in healthy controls; estimated premorbid IQ less than 65 based on the Wechsler Test of Adult Reading (WTAR); MRI contra-indicators; healthy subjects could not have a first degree relative with a psychotic illness. See Supplemental Figure S1 for additional inclusion and exclusion criteria.

**MRI data acquisition.** Neuroimaging data were collected on two identical 3T Phillips Intera Achieva MRI scanners equipped with a 32-channel head coil located at Vanderbilt Institute for Imaging Sciences (VUIIS). In all three studies (CT00762866; R01MH070560; R01MH102266) a high resolution T1-weighted anatomical scan was collected for each individual with a 3D T1 fast field echo sequence with a 1mm^3^ isotropic voxels (TR/TE = 8.0/3.7, FOV = 256 x 256 x 170, flip angle = 5°).

**Similarity network fusion.** The SNF approach has been previously described (Wang et al., 2014) and is publicly available (<http://compbio.cs.toronto.edu/SNF/SNF/Software.html>). SNF is a data integration approach that identifies participant similarity networks by integrating within and across data types (e.g., brain morphology, clinical), grouping participants who are most similar together. The fused network captures both shared and complementary information from different data types, providing insight into how informative each data type is to the observed similarity between samples.

This analysis creates separate networks for each data type (i.e., cortical thickness, surface area, and subcortical volume), describing participant similarity, followed by using a nonlinear combination method to iteratively fuse each data type network into a single participant similarity network, representing the full spectrum of included features. Across iterations (specified by hyperparameter T; default T=10-20), low-weight edges or similarities between participants in each data type are removed to reduce noise, while high-weight edges are added together. This is a key advantage of SNF over traditional clustering approaches, where the fusion step helps boost the signal to noise ratio if there is shared signal across data types. The similarity matrices are calculated using Euclidean distance, and two hyperparameters, a nearest neighbor value (K) and a normalization parameter (alpha). K indicates the number of neighbors of a node to consider when the similarity networks are being generated, smaller values will result in more sparsely connected networks, while larger values will generate denser networks. Alpha is the edge weighting parameter determining the weight of edges between nodes in the networks, smaller values will keep only the strongest edges, while larger values will retain a wider distribution of edge weights. The number of clusters is first estimated across 126 combinations of K and alpha hyperparameters (K=10-30; alpha=0.3-0.8), and a two-cluster solution was estimated to be the optimal number of clusters 126 out of 126 times as the best solution using eigen-gaps as well as separately for rotations costs. The consensus across iterations and alpha values estimated the number of clusters to be 2, so we selected a mid-range value of 0.6. K=30 was selected based on sample size (381/10=38.1). Another advantage of SNF is that it provides normalized mutual information (NMI) values, describing the overlap in a similarity matrix created using any single model feature, compared to the full spectrum fused matrix (NMI range 0-1). Higher NMI values indicate greater contribution to participant similarity, providing a rank of each model feature in the determination of data-driven clusters. Traditional clustering approaches also often rely on manual integration of data types and tend to suffer from issues to dimensionality, where data type with more features, regardless of their relative importance, dominate the resulting patient network [2]. SNF rather generates patient similarity networks separately for each data type and then iteratively fuses them together to create a single, multimodal patient network that reduces the likelihood of biasing results towards higher-dimensionality data. Finally, SNF is less complex to implement and allows for smaller sample sizes than recently developed semi-supervised machine learning methods such as HYDRA [3].

Within the SNF analysis, there is an embedded resampling-based stability testing (bootstrapping) with 1000 iterations. For each iteration, 80% of the subjects are first randomly sampled, then their features integrated using SNF, and finally clustered into subgroups and assigned a subtype label. For each iteration we can assess stability/consistency using three metrics. First, the consistency of the top contributing features based on NMI scores (Supplemental Figure 2). Second, the percent of time that each subject clustered with every other subject, when subjects were both included in the resampling iteration (Figure 2A, participant agreement matrix). Finally, the overlap between cluster partitions measured using the adjusted Rand index.

**Cell-type imputation**

*snRNA-seq data processing*

Reference single-nucleus RNA-sequencing (snRNA-seq) profiles were downloaded from Jorstad et al. [4]. Standard processing of unique molecular identifiers (UMIs) count matrices were performed in Seurat. The outlier cells and minimally expressed genes were excluded, after which the data were log-normalized. Only genes shared between Jorstad and Allen Human Brain Atlas (AHBA) datasets were included. To enhance specificity and computational efficiency, the gene sets were limited to robust marker genes across all the eight cortical areas in snRNA-seq samples. The 24-level cell type clusters from Jorstad et al. were used to categorize transcriptionally similar cell types to reduce collinearity and enhance interpretability. These 24 transcriptionally defined cell-types with featured electro-morphological properties (e.g., projecting style, cortical layer, firing rate, neurotransmitters) were found robust across cortical areas and different species [4]. Single cell samples across all eight cortical regions were combined into one gene-by-cell matrix then de-log-transformed. From the matrix, gene expression signature for each cell-type was derived via CIBERSORTx.

*Microarray data processing*

Microarray gene expression data from bulk tissue samples of six postmortem brains were downloaded from the AHBA [5]. Each sample was processed via abagen toolbox. Probes without Entrez ID or exceed background noise in less than 30% tissue samples were excluded, retaining the probes with the highest differential stability for each gene. Donor-wise expression data were normalized via a two-step z-score transformation (across genes, then across samples). To localize expression on the cortex, individual tissue samples were mapped to their donor's native FreeSurfer surface, which was subsequently registered to the fsLR32k group space. Samples located within 4 mm of the cortical surface were included.

*Cell-type deconvolution*

The cell-type gene expression signatures derived from snRNA and the total gene expression for each bulk tissue sample obtained from microarray data were fed into CIBERSORTx to decompose the fraction of each cell type within each tissue sample. The 24 cell-type fractions of each microarray sample were registered to the closest DK parcel based on the vertex ID of the sample. Parcel-level cell-type fractions were obtained by taking the average of samples registered to the same parcel.

**Clinical characteristics of psychosis subtypes.** No difference in average dosage of antipsychotic medication or duration of illness was found between subtypes. However, we conducted multiple linear regressions to examine if antipsychotic dose or duration of illness, separately, predict basal ganglia volumes (caudate, putamen, and globus pallidus), and whether this relationship changes across psychosis subtypes (controlling for ICV, age, biological sex, scan type, and scan quality (i.e., EFC)). Antipsychotic dose did not significantly predict basal ganglia volumes, nor did this relationship differ across psychosis subtypes after correcting for multiple comparisons (*p*_FDR_<0.05). However, regarding illness duration, longer illness duration was associated with larger left putamen volumes (*b*= 1.33, SE=0.48, *p*_FDR_=0.04) across subtypes.

Subtype 2 exhibited significantly higher PANSS negative symptom scores compared to subtype 1 (*b*=2.3, SE=0.7, *p*=0.001), but no difference in positive or general symptomatology (positive: *b*=0.99, SE=0.87, *p*=0.25; general: *b*=1.05, SE=0.95, *p*=0.27). Higher negative symptoms indicate greater severity of symptoms including social and emotional withdrawal, and blunted affect. Regarding cognition, subtype 2 displayed significantly lower WTAR predicted IQ scores compared to subtype 1 (*b*=-4.91, SE=1.02, *p*<0.001) and healthy controls (*b*=-9.46, SE=1.01, *p*<0.001). Subtype 1 had significantly lower WTAR predicted IQ scores compared to healthy controls (*b*=-4.55, SE=0.88, *p*<0.001). Lower WTAR scores indicate worse premorbid intellectual functioning. Similarly, subtype 2 displayed significantly lower SCIP composite *z*-scores compared to subtype 1 (*b*=-0.26, SE=0.08, *p*=0.002) and healthy controls (*b*=-1.22, SE=0.08, *p*<0.001). Subtype 1 had significantly lower SCIP composite *z*-scores compared to healthy controls (*b*=-0.96, SE=0.07, *p*<0.001). Lower SCIP scores indicate worse current neuropsychological functioning. Subtype 1 and subtype 2 did not display differences on premorbid adjustment (*b*=0.03, SE=0.09, *p*=0.75).

A hierarchical multiple regression was conducted to examine how much additional variance in negative symptoms, WTAR predicted IQ, and SCIP composite *z*-scores is explained by psychosis subtype beyond diagnosis and illness stage. In the first step, diagnosis (SSD, BD), illness stage (early, chronic), and biological sex accounted for 14% of the variance in negative symptoms (R^2^=0.14, *F*(3, 370)=21.12, *p*<0.001). Adding subtype in the second step significantly improved the model, explaining an additional 1.3% of the variance (R^2^=0.15, *F*(4, 369)=17.44, *p*<0.001). Regarding cognition, the test for the effect of psychosis subtype while controlling for diagnosis and illness stage is on one degree of freedom due to the controls having identical coding across the three factors (diagnosis, illness stage, subtype). Regarding premorbid IQ (WTAR), diagnosis, illness stage, and biological sex accounted for 14% of the variance (R^2^=0.14, *F*(4, 592)=26.01, *p*<0.001). Adding subtype in the second step significantly improved the model, explaining an additional 2.6% of the variance (R^2^=0.17, *F*(5, 591)=25.17, *p*<0.001). Regarding neuropsychological functioning (SCIP), diagnosis, illness stage, and biological sex accounted for 34% of the variance (R^2^=0.34, *F*(4, 606)=77.88, *p*<0.001). Adding subtype in the second step significantly improved the model, explaining an additional 1% of the variance (R^2^=0.34, *F*(5, 605)=64.22, *p*<0.001).

SUPPLEMENTAL FIGURES

**Figure S1.** **Subject inclusion/exclusion flow chart**. A series of steps for the final selection of participants in the current study. The final sample of 624 individuals included 243 healthy controls, 101 people with bipolar disorder with psychotic features, and 280 people with schizophrenia spectrum disorder. See Table 1 and Table S1 for sample demographics.

**Figure S2.** **Stability of the top 35 brain features contributing to data-driven psychosis subtypes**. The percentage of times that any given top contributing feature remained among the top 35 features across resampling was moderate to high.

**Figure S3.** **Unthresholded effect size brain maps of psychosis subtype 1 and 2 vs. healthy controls.** A) compared to healthy individuals, psychosis subtype 1 demonstrated patterns of primarily lower cortical thickness, larger surface area, and larger subcortical volumes. B) compared to healthy individuals, psychosis subtype 2 demonstrated patterns of primarily lower cortical thickness and surface area, and smaller subcortical volumes.

**Figure S4. Data-driven similarity network fusion analysis of brain morphology features revealed two clusters in SSD individuals.** Similarity network fusion (SNF) was separately conducted on a subset of individuals with a psychotic disorder, including only those with schizophrenia spectrum disorders (SSDs, n=280). Following identical procedures described in the main text methods, SNF and spectral clustering revealed two participant similarity network clusters (A) comprised of n=161 and n=119 individuals with SSD based on a consensus across eigen-gap and rotation costs. The average silhouette width was 0.93 indicating a strong cluster structure (A, silhouette plot).

Resampling-based stability testing with 1000 iterations, each using a random sampling of 80% of participants, revealed that clusters were highly stable. On average, 93% of the time any given participant clustered with each other participant within their data-driven group, while only 7% of the time an individual was clustered with a participant in another group (A, participant agreement matrix). The Adjusted Rand Index was 0.81 across 1000 iterations, indicating a high level of agreement between clustering solutions considering chance agreement.

Surface areas and subcortical volumes had higher NMI scores (B), indicating higher relative importance in determining clusters than thickness (comparable to results in the full psychosis cohort SNF results). The top 10 model features, based on NMI scores included subcortical volumes (caudate, hippocampus, thalamus) and surface area of the fusiform, precuneus, superior frontal, and medial orbitofrontal. No thickness features appeared in the top 10 or when the range was extended to the top 35.

Effect sizes (ESs) for the difference between SSD subtypes were calculated for all brain features to further illustrate the relative importance of regional brain features that differentiate the subtypes. C) Consistent with the NMI scores, ESs were moderate to large (ES ~0.4-1.3) for surface area, especially frontal, temporal, and midline areas, and subcortical volumes, whereas ESs were considerably smaller for thickness. Cool colors = greater reductions in cortical thickness, surface area, and subcortical volume in SSD subtype 2 vs. subtype 1, uncorrected for multiple comparisons.

**Figure S5.** **Data-driven SSD subtypes demonstrate notably similar neurostructural profiles (vs. healthy controls) as those identified in the full psychosis sample**. Statistical analyses comparing identified neurostructural subtypes only in SSD individuals followed identical procedures described in the main text methods. SSD subtype neurostructural profiles were established by comparing each subtype to healthy individuals. A) SSD subtype 1 demonstrated small to medium (ES=0.11 to 0.50) enlargement of surface areas in most cortical areas, lower thickness in inferior frontal and temporal regions, higher thickness in only the cuneus, and larger basal ganglia volumes (caudate, putamen, globus pallidum). In contrast, SSD subtype 2 was characterized by widespread smaller surface areas and lower thickness, and smaller subcortical volumes. ESs were notably larger for surface areas (ES=-0.31 to -1.05) and subcortical volumes (ES=-0.12 to -0.88) relative to thickness (ES=-0.69 to 0.14).

**Figure S6.** **Unthresholded effect size brain maps of SSD subtype 1 and 2 vs. healthy controls**. A) Compared to healthy individuals, SSD subtype 1 demonstrated a mixed pattern of higher (red) and lower (blue) cortical thickness, larger surface areas, and larger subcortical volumes. B) Compared to healthy individuals, SSD subtype 2 demonstrated patterns of primarily lower cortical thickness and surface area, and smaller subcortical volumes.

**Figure S7.** **Cortical thickness regions of interest, left hemisphere, density plots by psychosis subtype.** Representation of the distribution of cortical thickness values for each region of interest by psychosis subtype. *Z*-score values are standardized relative to the mean and standard deviation of the healthy controls.

**Figure S8.** **Cortical thickness regions of interest, right hemisphere, density plots by psychosis subtype.** Representation of the distribution of cortical thickness values for each region of interest by psychosis subtype. *Z*-score values are standardized relative to the mean and standard deviation of the healthy controls.

**Figure S9.** **Surface area regions of interest, left hemisphere, density plots by psychosis subtype.** Representation of the distribution of surface area values for each region of interest by psychosis subtype. *Z*-score values are standardized relative to the mean and standard deviation of the healthy controls.

**Figure S10.** **Surface area regions of interest, right hemisphere, density plots by psychosis subtype.** Representation of the distribution of surface area values for each region of interest by psychosis subtype. *Z*-score values are standardized relative to the mean and standard deviation of the healthy controls.

**Figure S11.** **Subcortical volume regions of interest density plots by psychosis subtype.** Representation of the distribution of subcortical volume values for each region of interest by psychosis subtype. *Z*-score values are standardized relative to the mean and standard deviation of the healthy controls.

SUPPLEMENTAL TABLES

**Table S1.** **Sample demographics and clinical characteristics by diagnostic category.**

|  | **Healthy Individuals**  (n=243) | **SSD**  (n=280) | **BD**  (n=101) | **Statistics** | | |
| --- | --- | --- | --- | --- | --- | --- |
|  | No. (%) | No. (%) | No. (%) | χ^2^ | df | *p*-value |
| **Sex** (female) | 91 (37.45) | 88 (31.43) | 49 (48.51) | 9.49 | 2 | *0.01* |
| **Race** |  |  |  | 23.03 | 8 | *0.005* |
| White | 169 (69.55) | 172 (61.43) | 80 (79.21) |  |  |  |
| Black | 54 (22.22) | 92 (32.86) | 12 (11.88) |  |  |  |
| Asian | 11 (4.53) | 5 (1.78) | 3 (2.97) |  |  |  |
| American Indian or Alaskan Native | 3 (1.23) | 2 (0.71) | 2 (1.98) |  |  |  |
| Other | 6 (2.47) | 9 (3.22) | 4 (3.96) |  |  |  |
| **Ethnicity** (Hispanic) | 12 (4.94) | 10 (3.57) | 7 (6.93) | 8.37 | 4 | 0.07 |
| **Handedness** (right) | 223 (91.77) | 249 (88.93) | 93 (92.08) | 1.25 | 2 | 0.53 |
| **Illness-Stage** (early/chronic) | --- | 179/101 | 57/43 |  |  |  |
|  |  |  |  |  |  |  |
|  | M ± SD | M ± SD | M ± SD | *F/t* | df | *p*-value |
| **Age** (years) | 28.03 ± 9.70 | 27.65 ± 10.32 | 30.36 ± 11.72 | 2.63 | 2 | 0.07 |
| **Education** (years) | 15.36 ± 2.11 | 13.41 ± 2.25 | 13.97 ± 1.94 | 52.28 | 2 | *<0.001 (C>BD/SSD)* |
| **Parental Education** (years) | 14.64 ± 2.29 | 14.63 ± 2.90 | 14.89 ± 2.26 | 0.37 | 2 | 0.69 |
| **Estimated Premorbid IQ** | 108.50 ± 7.57 | 100.88 ± 11.30 | 105.03 ± 9.05 | 39.28 | 2 | *<0.001 (C>BD>SSD)* |
| **SCIP** (z-score) | 0.14 ± 0.63 | -1 ± 0.94 | -0.67 ± 0.88 | 128.82 | 2 | *<0.001 (C>BD>SSD)* |
| **Age of Illness Onset** (years) | -- | 23.69 ± 8.45 | 21.00 ± 5.26 | 3.69 | 378 | *<0.001* |
| **Duration of Illness** (months) | --- | 74.71 ± 120.04 | 76.31 ± 108.68 | 0.12 | 378 | 0.91 |
| **PANSS Positive Symptoms** | --- | 18 ± 7.97 | 15.30 ± 8.93 | -2.77 | 372 | *0.005* |
| **PANSS Negative Symptoms** | --- | 15.96 ± 7.10 | 10.70 ± 3.87 | -6.91 | 372 | *<0.001* |
| **PANSS General Symptoms** | --- | 31.87 ± 9.21 | 26.98 ± 7.05 | -4.74 | 371 | *<0.001* |
| **Premorbid Adjustment** (total) | --- | 1.47 ± 0.67 | 1.09 ± 0.64 | -3.73 | 215 | *<0.001* |
| **CPZ Equivalents** | --- | 407.15 ± 551.70 | 292.01 ±210.26 | -1.78 | 318 | 0.08 |

**Note.** BD, bipolar with psychotic features; SSD, schizophrenia spectrum disorder; SCIP, Screen for Cognitive Impairment in Psychiatry; PANSS, Positive and Negative Syndrome Scale; Italics indicate significant *p*-values. Estimated premorbid IQ measured by the WTAR, Wechsler Test of Adult Reading predicted score. Duration of illness was defined as the time at which an individual first met criteria for psychosis (based on extensive interview, review of medical records, and collateral reports) until the date of study enrollment. CPZ, chlorpromazine equivalent.

**Table S2.** **Cortical thickness comparisons among patients and healthy controls.**

| Region | Cohen’s *d*  (PT v. HC) | SE | 95% CI | Estimate | *t* value | *p*-value | FDR  *p*-value |
| --- | --- | --- | --- | --- | --- | --- | --- |
| **L banks STS** | **-0.294** | **0.0831** | **[-0.46, -0.13]** | **-0.04** | **-3.56** | **0.0004***** | **0.001**** |
| **R banks STS** | **-0.341** | **0.0832** | **[-0.5, -0.18]** | **-0.05** | **-4.12** | **0.00004***** | **0.0003***** |
| L caudal anterior cingulate | -0.14 | 0.0827 | [-0.3, 0.02] | -0.03 | -1.70 | 0.09 | 0.11 |
| **R caudal anterior cingulate** | **-0.181** | **0.0828** | **[-0.34, -0.02]** | **-0.03** | **-2.19** | **0.03*** | **0.04*** |
| **L caudal middle frontal** | **-0.273** | **0.083** | **[-0.44, -0.11]** | **-0.04** | **-3.31** | **0.001**** | **0.003**** |
| **R caudal middle frontal** | **-0.231** | **0.0829** | **[-0.39, -0.07]** | **-0.03** | **-2.79** | **0.005**** | **0.01*** |
| L cuneus | -0.0339 | 0.0826 | [-0.2, 0.13] | 0.00 | -0.41 | 0.68 | 0.72 |
| R cuneus | -0.01 | 0.0826 | [-0.17, 0.15] | 0.00 | -0.12 | 0.9 | 0.9 |
| L entorhinal | 0.0129 | 0.0826 | [-0.15, 0.18] | 0.00 | 0.16 | 0.88 | 0.9 |
| R entorhinal | -0.164 | 0.0828 | [-0.33, 0] | -0.05 | -1.98 | 0.048* | 0.07 |
| **L fusiform** | **-0.323** | **0.0831** | **[-0.49, -0.16]** | **-0.04** | **-3.91** | **0.0001**** | **0.0004***** |
| **R fusiform** | **-0.263** | **0.083** | **[-0.43, -0.1]** | **-0.04** | **-3.18** | **0.002**** | **0.004**** |
| **L inferior parietal** | **-0.241** | **0.0829** | **[-0.4, -0.08]** | **-0.03** | **-2.92** | **0.004**** | **0.008**** |
| **R inferior parietal** | **-0.342** | **0.0832** | **[-0.51, -0.18]** | **-0.04** | **-4.13** | **0.00004***** | **0.0003***** |
| L inferior temporal | -0.153 | 0.0827 | [-0.32, 0.01] | -0.02 | -1.85 | 0.064 | 0.09 |
| **R inferior temporal** | **-0.239** | **0.0829** | **[-0.4, -0.08]** | **-0.03** | **-2.89** | **0.004**** | **0.009**** |
| L isthmus cingulate | -0.0503 | 0.0826 | [-0.21, 0.11] | -0.01 | -0.61 | 0.54 | 0.6 |
| R isthmus cingulate | -0.0825 | 0.0827 | [-0.24, 0.08] | -0.01 | -1.00 | 0.32 | 0.37 |
| **L lateral occipital** | **-0.293** | **0.0831** | **[-0.46, -0.13]** | **-0.03** | **-3.55** | **0.0004***** | **0.001**** |
| **R lateral occipital** | **-0.296** | **0.0831** | **[-0.46, -0.13]** | **-0.03** | **-3.58** | **0.0004***** | **0.001**** |
| **L lateral orbitofrontal** | **-0.175** | **0.0828** | **[-0.34, -0.01]** | **-0.03** | **-2.12** | **0.03*** | **0.049*** |
| **R lateral orbitofrontal** | **-0.216** | **0.0829** | **[-0.38, -0.05]** | **-0.03** | **-2.61** | **0.009**** | **0.02*** |
| L lingual | -0.145 | 0.0827 | [-0.31, 0.02] | -0.02 | -1.75 | 0.08 | 0.11 |
| R lingual | -0.149 | 0.0827 | [-0.31, 0.01] | -0.02 | -1.81 | 0.07 | 0.09 |
| L medial orbitofrontal | -0.134 | 0.0827 | [-0.3, 0.03] | -0.02 | -1.62 | 0.11 | 0.13 |
| R medial orbitofrontal | -0.131 | 0.0827 | [-0.29, 0.03] | -0.01 | -1.59 | 0.11 | 0.13 |
| **L middle temporal** | **-0.308** | **0.0831** | **[-0.47, -0.15]** | **-0.04** | **-3.73** | **0.0002***** | **0.0008***** |
| **R middle temporal** | **-0.346** | **0.0832** | **[-0.51, -0.18]** | **-0.05** | **-4.19** | **0.00003***** | **0.0003***** |
| **L parahippocampal** | **-0.251** | **0.0829** | **[-0.41, -0.09]** | **-0.06** | **-3.04** | **0.003**** | **0.006**** |
| **R parahippocampal** | **-0.184** | **0.0828** | **[-0.35, -0.02]** | **-0.04** | **-2.23** | **0.03*** | **0.04*** |
| **L paracentral** | **-0.238** | **0.0829** | **[-0.4, -0.08]** | **-0.03** | **-2.88** | **0.004**** | **0.009**** |
| **R paracentral** | **-0.178** | **0.0828** | **[-0.34, -0.02]** | **-0.03** | **-2.16** | **0.03*** | **0.047*** |
| **L pars opercularis** | **-0.415** | **0.0835** | **[-0.58, -0.25]** | **-0.06** | **-5.02** | **<0.000001***** | **0.00002***** |
| **R pars opercularis** | **-0.223** | **0.0829** | **[-0.39, -0.06]** | **-0.03** | **-2.69** | **0.007**** | **0.01*** |
| **L pars orbitalis** | **-0.246** | **0.0829** | **[-0.41, -0.08]** | **-0.05** | **-2.97** | **0.003**** | **0.007**** |
| **R pars orbitalis** | **-0.267** | **0.083** | **[-0.43, -0.1]** | **-0.05** | **-3.23** | **0.001**** | **0.003**** |
| **L pars triangularis** | **-0.337** | **0.0832** | **[-0.5, -0.17]** | **-0.05** | **-4.07** | **0.00005***** | **0.0003***** |
| **R pars triangularis** | **-0.337** | **0.0832** | **[-0.5, -0.17]** | **-0.05** | **-4.08** | **0.00005***** | **0.0003***** |
| L pericalcarine | 0.0498 | 0.0826 | [-0.11, 0.21] | 0.01 | 0.60 | 0.55 | 0.6 |
| R pericalcarine | 0.029 | 0.0826 | [-0.13, 0.19] | 0.00 | 0.35 | 0.73 | 0.76 |
| **L postcentral** | **-0.218** | **0.0829** | **[-0.38, -0.06]** | **-0.02** | **-2.64** | **0.009**** | **0.02*** |
| **R postcentral** | **-0.224** | **0.0829** | **[-0.39, -0.06]** | **-0.02** | **-2.71** | **0.007**** | **0.01*** |
| L posterior cingulate | -0.134 | 0.0827 | [-0.3, 0.03] | -0.02 | -1.62 | 0.11 | 0.13 |
| **R posterior cingulate** | **-0.223** | **0.0829** | **[-0.39, -0.06]** | **-0.03** | **-2.69** | **0.007**** | **0.01*** |
| **L precentral** | **-0.401** | **0.0834** | **[-0.56, -0.24]** | **-0.05** | **-4.85** | **<0.000001***** | **0.00002***** |
| **R precentral** | **-0.324** | **0.0831** | **[-0.49, -0.16]** | **-0.04** | **-3.92** | **0.0001***** | **0.0004***** |
| **L precuneus** | **-0.218** | **0.0829** | **[-0.38, -0.06]** | **-0.03** | **-2.63** | **0.009**** | **0.02*** |
| **R precuneus** | **-0.223** | **0.0829** | **[-0.39, -0.06]** | **-0.03** | **-2.69** | **0.007**** | **0.01*** |
| **L rostral anterior cingulate** | **-0.199** | **0.0828** | **[-0.36, -0.04]** | **-0.03** | **-2.41** | **0.02*** | **0.03*** |
| R rostral anterior cingulate | 0.0445 | 0.0826 | [-0.12, 0.21] | 0.01 | 0.54 | 0.59 | 0.64 |
| **L rostral middle frontal** | **-0.318** | **0.0831** | **[-0.48, -0.16]** | **-0.04** | **-3.84** | **0.0001***** | **0.0005***** |
| **R rostral middle frontal** | **-0.334** | **0.0832** | **[-0.5, -0.17]** | **-0.04** | **-4.05** | **0.00006***** | **0.0003***** |
| **L superior frontal** | **-0.342** | **0.0832** | **[-0.51, -0.18]** | **-0.05** | **-4.14** | **0.00004***** | **0.0003***** |
| **R superior frontal** | **-0.33** | **0.0832** | **[-0.49, -0.17]** | **-0.05** | **-3.99** | **0.00007***** | **0.0003***** |
| L superior parietal | -0.144 | 0.0827 | [-0.31, 0.02] | -0.02 | -1.74 | 0.08 | 0.11 |
| **R superior parietal** | **-0.178** | **0.0828** | **[-0.34, -0.02]** | **-0.02** | **-2.16** | **0.03*** | **0.047*** |
| **L superior temporal** | **-0.342** | **0.0832** | **[-0.51, -0.18]** | **-0.05** | **-4.14** | **0.00004***** | **0.0003***** |
| **R superior temporal** | **-0.402** | **0.0834** | **[-0.57, -0.24]** | **-0.06** | **-4.86** | **<0.000001***** | **0.00002***** |
| **L supramarginal** | **-0.349** | **0.0832** | **[-0.51, -0.19]** | **-0.04** | **-4.22** | **0.00003***** | **0.0003***** |
| **R supramarginal** | **-0.315** | **0.0831** | **[-0.48, -0.15]** | **-0.04** | **-3.81** | **0.0002***** | **0.0006***** |
| **L frontal pole** | **-0.275** | **0.083** | **[-0.44, -0.11]** | **-0.07** | **-3.33** | **0.0009***** | **0.003**** |
| R frontal pole | -0.0107 | 0.0826 | [-0.17, 0.15] | 0.00 | -0.13 | 0.89 | 0.9 |
| L temporal pole | -0.0777 | 0.0827 | [-0.24, 0.08] | -0.02 | -0.94 | 0.35 | 0.39 |
| R temporal pole | -0.0798 | 0.0827 | [-0.24, 0.08] | -0.03 | -0.97 | 0.33 | 0.39 |
| **L transverse temporal** | **-0.283** | **0.083** | **[-0.45, -0.12]** | **-0.06** | **-3.43** | **0.0007***** | **0.002**** |
| **R transverse temporal** | **-0.383** | **0.0834** | **[-0.55, -0.22]** | **-0.08** | **-4.63** | **<0.000001***** | **0.00002***** |
| **L insula** | **-0.197** | **0.0828** | **[-0.36, -0.03]** | **-0.03** | **-2.39** | **0.02*** | **0.03*** |
| R insula | -0.159 | 0.0828 | [-0.32, 0] | -0.03 | -1.93 | 0.05 | 0.08 |

**Note.** General linear models per region controlling for age, sex, scan version, and T1w quality (MRIQC efc). **p*<0.05, ***p*<0.01, ****p*<0.001. FDR corrected using Benjamini-Hochberg procedure across all CT regions. Regions with *p*_FDR_<0.05 are depicted in bold. PT = patient, HC = healthy control, SE = standard error, L = left, R = right.

**Table S3.** **Surface area comparisons among patients and healthy controls.**

| Region | Cohen’s *d*  (PT v. HC) | SE | 95% CI | Estimate | *t* value | *p*-value | FDR  *p*-value |
| --- | --- | --- | --- | --- | --- | --- | --- |
| L banks STS | -0.107 | 0.0827 | [-0.27, 0.06] | -17.71 | -1.29 | 0.197 | 0.974 |
| R banks STS | -0.0596 | 0.0827 | [-0.22, 0.1] | -7.71 | -0.72 | 0.471 | 0.979 |
| L caudal anterior cingulate | -0.0353 | 0.0826 | [-0.2, 0.13] | -4.71 | -0.43 | 0.669 | 0.979 |
| R caudal anterior cingulate | -0.0917 | 0.0827 | [-0.25, 0.07] | -13.82 | -1.11 | 0.268 | 0.974 |
| L caudal middle frontal | -0.0626 | 0.0827 | [-0.23, 0.1] | -22.13 | -0.76 | 0.449 | 0.979 |
| R caudal middle frontal | -0.0183 | 0.0826 | [-0.18, 0.14] | -6.46 | -0.22 | 0.825 | 0.979 |
| L cuneus | -0.0138 | 0.0826 | [-0.18, 0.15] | -2.94 | -0.17 | 0.867 | 0.979 |
| R cuneus | 0.051 | 0.0826 | [-0.11, 0.21] | 10.72 | 0.62 | 0.537 | 0.979 |
| L entorhinal | -0.102 | 0.0827 | [-0.26, 0.06] | -9.31 | -1.23 | 0.217 | 0.974 |
| R entorhinal | -0.116 | 0.0827 | [-0.28, 0.05] | -9.39 | -1.40 | 0.161 | 0.974 |
| L fusiform | -0.114 | 0.0827 | [-0.28, 0.05] | -40.92 | -1.38 | 0.168 | 0.974 |
| R fusiform | -0.184 | 0.0828 | [-0.35, -0.02] | -60.18 | -2.23 | 0.026* | 0.442 |
| L inferior parietal | -0.12 | 0.0827 | [-0.28, 0.04] | -71.37 | -1.45 | 0.148 | 0.974 |
| R inferior parietal | -0.158 | 0.0828 | [-0.32, 0.004] | -111.73 | -1.91 | 0.056 | 0.762 |
| L inferior temporal | -0.189 | 0.0828 | [-0.35, -0.03] | -89.15 | -2.28 | 0.023* | 0.442 |
| R inferior temporal | -0.129 | 0.0827 | [-0.29, 0.03] | -54.62 | -1.56 | 0.119 | 0.974 |
| L isthmus cingulate | 0.0188 | 0.0826 | [-0.14, 0.18] | 2.88 | 0.23 | 0.820 | 0.979 |
| R isthmus cingulate | 0.0048 | 0.0826 | [-0.16, 0.17] | 0.63 | 0.06 | 0.954 | 0.986 |
| L lateral occipital | -0.0028 | 0.0826 | [-0.17, 0.16] | -1.65 | -0.03 | 0.973 | 0.986 |
| R lateral occipital | -0.106 | 0.0827 | [-0.27, 0.06] | -64.97 | -1.28 | 0.200 | 0.974 |
| L lateral orbitofrontal | -0.0411 | 0.0826 | [-0.2, 0.12] | -12.59 | -0.50 | 0.619 | 0.979 |
| R lateral orbitofrontal | -0.0947 | 0.0827 | [-0.26, 0.07] | -32.29 | -1.15 | 0.252 | 0.974 |
| L lingual | -0.0768 | 0.0827 | [-0.24, 0.09] | -30.56 | -0.93 | 0.353 | 0.979 |
| R lingual | -0.0878 | 0.0827 | [-0.25, 0.07] | -36.26 | -1.06 | 0.288 | 0.979 |
| L medial orbitofrontal | -0.0652 | 0.0827 | [-0.23, 0.1] | -14.34 | -0.79 | 0.430 | 0.979 |
| R medial orbitofrontal | 0.0128 | 0.0826 | [-0.15, 0.18] | 2.78 | 0.16 | 0.877 | 0.979 |
| L middle temporal | -0.209 | 0.0828 | [-0.37, -0.05] | -85.59 | -2.53 | 0.012* | 0.394 |
| R middle temporal | -0.151 | 0.0827 | [-0.31, 0.01] | -66.62 | -1.83 | 0.067 | 0.763 |
| L parahippocampal | -0.0559 | 0.0826 | [-0.22, 0.11] | -4.00 | -0.68 | 0.499 | 0.979 |
| R parahippocampal | -0.0271 | 0.0826 | [-0.19, 0.14] | -2.07 | -0.33 | 0.743 | 0.979 |
| L paracentral | -0.0495 | 0.0826 | [-0.21, 0.11] | -8.04 | -0.60 | 0.549 | 0.979 |
| R paracentral | -0.0626 | 0.0827 | [-0.23, 0.1] | -11.20 | -0.76 | 0.449 | 0.979 |
| L pars opercularis | 0.0466 | 0.0826 | [-0.12, 0.21] | 11.79 | 0.56 | 0.573 | 0.979 |
| R pars opercularis | -0.0358 | 0.0826 | [-0.2, 0.13] | -7.15 | -0.43 | 0.665 | 0.979 |
| L pars orbitalis | -0.0965 | 0.0827 | [-0.26, 0.07] | -9.11 | -1.17 | 0.243 | 0.974 |
| R pars orbitalis | -0.0312 | 0.0826 | [-0.19, 0.13] | -3.49 | -0.38 | 0.706 | 0.979 |
| L pars triangularis | 0.0015 | 0.0826 | [-0.16, 0.16] | 0.28 | 0.02 | 0.986 | 0.986 |
| R pars triangularis | -0.0127 | 0.0826 | [-0.18, 0.15] | -2.88 | -0.15 | 0.878 | 0.979 |
| L pericalcarine | 0.0141 | 0.0826 | [-0.15, 0.18] | 3.60 | 0.17 | 0.865 | 0.979 |
| R pericalcarine | 0.0027 | 0.0826 | [-0.16, 0.17] | 0.70 | 0.03 | 0.974 | 0.986 |
| L postcentral | -0.0834 | 0.0827 | [-0.25, 0.08] | -37.99 | -1.01 | 0.313 | 0.979 |
| R postcentral | -0.0318 | 0.0826 | [-0.19, 0.13] | -13.91 | -0.38 | 0.701 | 0.979 |
| L posterior cingulate | -0.111 | 0.0827 | [-0.27, 0.05] | -18.15 | -1.34 | 0.181 | 0.974 |
| R posterior cingulate | -0.0341 | 0.0826 | [-0.2, 0.13] | -5.72 | -0.41 | 0.680 | 0.979 |
| L precentral | -0.0762 | 0.0827 | [-0.24, 0.09] | -39.21 | -0.92 | 0.357 | 0.979 |
| R precentral | -0.0476 | 0.0826 | [-0.21, 0.12] | -22.51 | -0.58 | 0.564 | 0.979 |
| L precuneus | -0.0052 | 0.0826 | [-0.17, 0.16] | -2.42 | -0.06 | 0.950 | 0.986 |
| R precuneus | -0.0296 | 0.0826 | [-0.19, 0.13] | -14.41 | -0.36 | 0.720 | 0.979 |
| L rostral anterior cingulate | -0.0501 | 0.0826 | [-0.21, 0.11] | -8.23 | -0.61 | 0.545 | 0.979 |
| R rostral anterior cingulate | -0.23 | 0.0829 | [-0.39, -0.07] | -29.46 | -2.78 | 0.006** | 0.374 |
| L rostral middle frontal | -0.0178 | 0.0826 | [-0.18, 0.14] | -12.31 | -0.22 | 0.829 | 0.979 |
| R rostral middle frontal | -0.002 | 0.0826 | [-0.16, 0.16] | -1.49 | -0.02 | 0.981 | 0.986 |
| L superior frontal | -0.0454 | 0.0826 | [-0.21, 0.12] | -37.45 | -0.55 | 0.583 | 0.979 |
| R superior frontal | -0.0908 | 0.0827 | [-0.25, 0.07] | -74.66 | -1.10 | 0.272 | 0.974 |
| L superior parietal | -0.0144 | 0.0826 | [-0.18, 0.15] | -9.90 | -0.17 | 0.861 | 0.979 |
| R superior parietal | -0.0289 | 0.0826 | [-0.19, 0.13] | -17.99 | -0.35 | 0.727 | 0.979 |
| L superior temporal | -0.0425 | 0.0826 | [-0.21, 0.12] | -18.97 | -0.51 | 0.607 | 0.979 |
| R superior temporal | -0.0608 | 0.0827 | [-0.22, 0.1] | -23.60 | -0.74 | 0.462 | 0.979 |
| L supramarginal | -0.0356 | 0.0826 | [-0.2, 0.13] | -22.82 | -0.43 | 0.667 | 0.979 |
| R supramarginal | 0.0551 | 0.0826 | [-0.11, 0.22] | 27.20 | 0.67 | 0.505 | 0.979 |
| L frontal pole | 0.0303 | 0.0826 | [-0.13, 0.19] | 1.06 | 0.37 | 0.714 | 0.979 |
| R frontal pole | -0.0181 | 0.0826 | [-0.18, 0.14] | -0.81 | -0.22 | 0.827 | 0.979 |
| L temporal pole | 0.0021 | 0.0826 | [-0.16, 0.16] | 0.14 | 0.03 | 0.980 | 0.986 |
| R temporal pole | -0.0467 | 0.0826 | [-0.21, 0.12] | -3.24 | -0.56 | 0.572 | 0.979 |
| L transverse temporal | -0.0163 | 0.0826 | [-0.18, 0.15] | -1.06 | -0.20 | 0.844 | 0.979 |
| R transverse temporal | 0.0132 | 0.0826 | [-0.15, 0.18] | 0.60 | 0.16 | 0.873 | 0.979 |
| L insula | -0.0956 | 0.0827 | [-0.26, 0.07] | -26.64 | -1.16 | 0.248 | 0.974 |
| R insula | -0.0685 | 0.0827 | [-0.23, 0.09] | -15.78 | -0.83 | 0.407 | 0.979 |

**Note.** General linear models per region controlling for age, sex, scan version, and T1w quality (MRIQC efc). **p*<0.05, ***p*<0.01, ****p*<0.001. FDR corrected using Benjamini-Hochberg procedure across all SA regions. Regions with *p*_FDR_<0.05 are depicted in bold. PT = patient, HC = healthy control, SE = standard error, L = left, R = right.

**Table S4.** **Subcortical volume comparisons among patients and healthy controls.**

| Region | Cohen’s *d*  (PT v. HC) | SE | 95% CI | Estimate | *t* value | *p*-value | FDR  *p*-value |
| --- | --- | --- | --- | --- | --- | --- | --- |
| **L thalamus** | **-0.291** | **0.0831** | **[-0.45, -0.13]** | **-164.67** | **-3.52** | **0.00047***** | **0.0013**** |
| **R thalamus** | **-0.298** | **0.0831** | **[-0.46, 0.14]** | **-154.61** | **-3.60** | **0.00034***** | **0.0012**** |
| L caudate | 0.168 | 0.0828 | [0.01, 0.33] | 67.01 | 2.03 | 0.04 | 0.062 |
| R caudate | 0.139 | 0.0827 | [-0.02, 0.30] | 56.32 | 1.68 | 0.09 | 0.13 |
| L putamen | 0.021 | 0.0826 | [-0.41, 0.18] | 12.82 | 0.26 | 0.79 | 0.79 |
| R putamen | 0.18 | 0.0828 | [0.02, 0.34] | 80.82 | 2.18 | 0.03* | 0.06 |
| L pallidum | 0.08 | 0.0827 | [-0.08, 0.24] | 16.97 | 0.97 | 0.33 | 0.39 |
| R pallidum | 0.071 | 0.0827 | [-0.09, 0.23] | 13.39 | 0.86 | 0.39 | 0.42 |
| **L hippocampus** | **-0.339** | **0.0832** | **[-0.50, -0.18]** | **-111.44** | **-4.10** | **0.00005***** | **0.0007***** |
| **R hippocampus** | **-0.303** | **0.0831** | **[-0.47, -0.14]** | **-100.27** | **-3.67** | **0.00027***** | **0.0012**** |
| **L amygdala** | **-0.246** | **0.0829** | **[-0.41, -0.08]** | **-50.87** | **-2.97** | **0.003**** | **0.007**** |
| **R amygdala** | **-0.322** | **0.0831** | **[-0.49, -0.16]** | **-68.49** | **-3.89** | **0.0001***** | **0.0007***** |
| L accumbens | -0.169 | 0.0828 | [-0.33, -0.01] | -17.35 | -2.04 | 0.04* | 0.062 |
| R accumbens | -0.116 | 0.0827 | [-0.28, 0.05] | -10.24 | -1.39 | 0.16 | 0.20 |

**Note.** General linear models per region controlling for age, sex, scan version, T1w quality (MRIQC efc), and intracranial volume. **p*<0.05, ***p*<0.01, ****p*<0.001. FDR corrected using Benjamini-Hochberg procedure across all SV regions. Regions with *p*_FDR_<0.05 are depicted in bold. PT = patient, HC = healthy control, SE = standard error, L = left, R = right.

**Table S5.** **Sample demographics and clinical characteristics by data-driven psychosis subtype.**

|  | **Healthy Individuals**  (n=243) | **Subtype 1**  (n=233) | **Subtype 2**  (n=148) | **Statistics** | | |
| --- | --- | --- | --- | --- | --- | --- |
|  | No. (%) | No. (%) | No. (%) | χ^2^ | df | *p*-value |
| **Sex** (female) | 91 (37.45) | 87 (37.34) | 50 (33.78) | 0.64 | 2 | 0.73 |
| **Race** |  |  |  | 57.65 | 8 | *<0.001* |
| White | 169 (69.55) | 185 (79.40) | 67 (45.27) |  |  |  |
| Black | 54 (22.22) | 36 (15.45) | 68 (45.95) |  |  |  |
| Asian | 11 (4.53) | 2 (0.89) | 6 (4.05) |  |  |  |
| American Indian or Alaskan Native | 3 (1.23) | 3 (1.29) | 1 (0.68) |  |  |  |
| Other | 6 (2.47) | 7 (3.00) | 6 (4.05) |  |  |  |
| **Ethnicity** (Hispanic) | 12 (4.94) | 13 (5.58) | 4 (2.70) | 8.05 | 4 | 0.09 |
| **Handedness** (right) | 223 (91.77) | 214 (91.85) | 128 (86.49) | 4.09 | 2 | 0.13 |
| **Diagnosis** (SSD/BD) | --- | 158/75 | 122/26 | 9.93 | 1 | *0.002* |
| **Illness-Stage** (early/chronic) | --- | 141/91 | 95/53 | 0.31 | 1 | 0.58 |
|  |  |  |  |  |  |  |
|  | M ± SD | M ± SD | M ± SD | *F/t* | df | *p*-value |
| **Age** (years) | 28.03 ± 9.70 | 28.66 ± 11.06 | 27.90 ± 10.30 | 0.32 | 2 | 0.72 |
| **Education** (years) | 15.36 ± 2.11 | 13.60 ± 2.20 | 13.47 ± 2.16 | 49.73 | 2 | *<0.001*  *(C>S1/S2)* |
| **Parental Education** (years) | 14.64 ± 2.29 | 14.83 ± 2.75 | 14.49 ± 2.76 | 0.71 | 2 | 0.49 |
| **Estimated Premorbid IQ** | 108.50 ± 7.57 | 103.86 ± 10.17 | 99.04 ± 11.37 | 44.87* | 2 | *<0.001* |
| **SCIP** (*z*-score) | 0.14 ± 0.63 | -0.82 ± 0.89 | -1.07 ± 0.98 | 137.23* | 2 | *<0.001* |
| **Age of Illness Onset** (years) | --- | 21.72 ± 6.61 | 21.70 ± 5.95 | 0.04 | 378 | 0.97 |
| **Duration of Illness** (months) | --- | 79.28 ± 121.55 | 68.62 ± 109.62 | 0.87 | 378 | 0.39 |
| **PANSS Positive Symptoms** | --- | 16.91 ± 8.32 | 17.93 ± 8.25 | 1.14* | 373 | 0.25 |
| **PANSS Negative Symptoms** | --- | 13.67 ± 6.17 | 16.05 ± 7.52 | 3.27* | 373 | *0.001* |
| **PANSS General Symptoms** | --- | 30.19 ± 8.75 | 31.28 ± 9.26 | 1.11* | 372 | 0.27 |
| **Premorbid Adjustment** (total) | --- | 1.35 ± 0.71 | 1.40 ± 0.64 | 0.32* | 216 | 0.75 |
| **CPZ Equivalents** | --- | 400.08 ± 588.37 | 343.67 ± 251.19 | 0.98 | 318 | 0.33 |

**Note.** SSD, Schizophrenia Spectrum Disorder; BD, Bipolar with Psychotic Features. Duration of illness was defined as the time at which an individual first met criteria for psychosis (based on extensive interview, review of medical records, and collateral reports) until the date of study enrollment. One patient in subtype 1 missing illness-stage data. Group differences on estimated premorbid IQ measured by the WTAR, Wechsler Test of Adult Reading predicted score; SCIP, Screen for Cognitive Impairment in Psychiatry; PANSS, Positive and Negative Syndrome Scale; and Premorbid Adjustment (n=217) were tested using separate general linear models (denoted with *, *t*- and *F*-values using the *aov* package in R [version 4.4.1] displayed here for consistency), detailed in the main text and Figure 4. Italics indicate significant *p*-values.

**Table S6. Normalized mutual information (NMI) values for all in-model brain features.**

| Rank | Model Brain Feature | NMI | Rank | Model Brain Feature | NMI |
| --- | --- | --- | --- | --- | --- |
| 1 | R hippocampus | 0.258 | 76 | L frontal pole, SA | 0.045 |
| 2 | L amygdala | 0.219 | 77 | L pars opercularis, SA | 0.043 |
| 3 | L hippocampus | 0.212 | 78 | L pars triangularis, SA | 0.039 |
| 4 | R amygdala | 0.194 | 79 | L entorhinal, SA | 0.039 |
| 5 | L medial orbitofrontal, SA | 0.191 | 80 | R pericalcarine, SA | 0.039 |
| 6 | R inferior temporal, SA | 0.188 | 81 | L temporal pole, SA | 0.038 |
| 7 | L precuneus, SA | 0.186 | 82 | R temporal pole, SA | 0.038 |
| 8 | R precuneus, SA | 0.185 | 83 | R precentral, CT | 0.036 |
| 9 | L precentral, SA | 0.177 | 84 | R pericalcarine, CT | 0.035 |
| 10 | L thalamus | 0.174 | 85 | L lingual, CT | 0.034 |
| 11 | R fusiform, SA | 0.169 | 86 | R frontal pole, SA | 0.034 |
| 12 | R thalamus | 0.166 | 87 | R inferior parietal, CT | 0.032 |
| 13 | R middle temporal, SA | 0.163 | 88 | R caudal middle frontal, CT | 0.032 |
| 14 | L inferior temporal, SA | 0.160 | 89 | R lateral occipital, CT | 0.031 |
| 15 | L superior parietal, SA | 0.155 | 90 | L caudal anterior cingulate, SA | 0.031 |
| 16 | L postcentral, SA | 0.152 | 91 | L postcentral, CT | 0.031 |
| 17 | R posterior cingulate, SA | 0.152 | 92 | L precuneus, CT | 0.029 |
| 18 | L caudate | 0.150 | 93 | L posterior cingulate, CT | 0.029 |
| 19 | L rostral middle frontal, SA | 0.149 | 94 | L banks sts, CT | 0.029 |
| 20 | R rostral middle frontal, SA | 0.149 | 95 | R cuneus, CT | 0.028 |
| 21 | L middle temporal, SA | 0.145 | 96 | R supramarginal, CT | 0.026 |
| 22 | L superior frontal, SA | 0.143 | 97 | L inferior parietal, CT | 0.026 |
| 23 | L superior temporal, SA | 0.142 | 98 | L fusiform, CT | 0.023 |
| 24 | R medial orbitofrontal, SA | 0.141 | 99 | R pars opercularis, CT | 0.022 |
| 25 | R superior parietal, SA | 0.140 | 100 | R lingual, CT | 0.021 |
| 26 | R caudate | 0.139 | 101 | L superior parietal, CT | 0.021 |
| 27 | L fusiform, SA | 0.139 | 102 | R superior parietal, CT | 0.019 |
| 28 | R superior frontal, SA | 0.135 | 103 | L cuneus, CT | 0.019 |
| 29 | R precentral, SA | 0.129 | 104 | R precuneus, CT | 0.019 |
| 30 | L putamen | 0.126 | 105 | R paracentral, CT | 0.019 |
| 31 | R postcentral, SA | 0.126 | 106 | R superior temporal, CT | 0.018 |
| 32 | R supramarginal, SA | 0.125 | 107 | L superior temporal, CT | 0.017 |
| 33 | L lateral orbitofrontal, SA | 0.122 | 108 | L paracentral, CT | 0.016 |
| 34 | L pars orbitalis, SA | 0.119 | 109 | L precentral, CT | 0.015 |
| 35 | R superior temporal, SA | 0.116 | 110 | R fusiform, CT | 0.015 |
| 36 | R paracentral, SA | 0.116 | 111 | R superior frontal, CT | 0.013 |
| 37 | R accumbens | 0.116 | 112 | L middle temporal, CT | 0.012 |
| 38 | R putamen | 0.115 | 113 | R insula, CT | 0.011 |
| 39 | L inferior parietal, SA | 0.113 | 114 | R inferior temporal, CT | 0.011 |
| 40 | L paracentral, SA | 0.111 | 115 | R transverse temporal, CT | 0.010 |
| 41 | R lateral orbitofrontal, SA | 0.110 | 116 | L rostral middle frontal, CT | 0.010 |
| 42 | R inferior parietal, SA | 0.110 | 117 | R middle temporal, CT | 0.009 |
| 43 | L lateral occipital, SA | 0.107 | 118 | L isthmus cingulate, CT | 0.009 |
| 44 | L posterior cingulate, SA | 0.106 | 119 | L transverse temporal, CT | 0.009 |
| 45 | L rostral anterior cingulate, SA | 0.105 | 120 | R rostral middle frontal, CT | 0.008 |
| 46 | L cuneus, SA | 0.102 | 121 | R banks sts, CT | 0.008 |
| 47 | L isthmus cingulate, SA | 0.098 | 122 | L caudal anterior cingulate, CT | 0.008 |
| 48 | R pars orbitalis, SA | 0.097 | 123 | R lateral orbitofrontal, CT | 0.007 |
| 49 | L transverse temporal, SA | 0.096 | 124 | R isthmus cingulate, CT | 0.006 |
| 50 | R isthmus cingulate, SA | 0.091 | 125 | R pars triangularis, CT | 0.006 |
| 51 | L lingual, SA | 0.091 | 126 | L caudal middle frontal, CT | 0.005 |
| 52 | L supramarginal, SA | 0.091 | 127 | R posterior cingulate, CT | 0.005 |
| 53 | L insula, SA | 0.089 | 128 | L superior frontal, CT | 0.005 |
| 54 | L pallidum | 0.086 | 129 | L inferior temporal, CT | 0.004 |
| 55 | R rostral anterior cingulate, SA | 0.083 | 130 | L supramarginal, CT | 0.003 |
| 56 | R lateral occipital, SA | 0.083 | 131 | L insula, CT | 0.003 |
| 57 | L caudal middle frontal, SA | 0.082 | 132 | R parahippocampal, CT | 0.003 |
| 58 | R insula, SA | 0.080 | 133 | L entorhinal, CT | 0.002 |
| 59 | R pars triangularis, SA | 0.079 | 134 | L pars orbitalis, CT | 0.002 |
| 60 | R banks sts, SA | 0.079 | 135 | L lateral orbitofrontal, CT | 0.002 |
| 61 | L pericalcarine, SA | 0.077 | 136 | L medial orbitofrontal, CT | 0.001 |
| 62 | R pars opercularis, SA | 0.076 | 137 | R medial orbitofrontal, CT | 0.001 |
| 63 | R pallidum | 0.075 | 138 | L pericalcarine, CT | 0.001 |
| 64 | R lingual, SA | 0.075 | 139 | R frontal pole, CT | 0.001 |
| 65 | R cuneus, SA | 0.071 | 140 | L pars opercularis, CT | 0.001 |
| 66 | R caudal middle frontal, SA | 0.070 | 141 | R temporal pole, CT | 0.001 |
| 67 | L accumbens | 0.069 | 142 | L rostral anterior cingulate, CT | 0.000 |
| 68 | R transverse temporal, SA | 0.067 | 143 | R caudal anterior cingulate, CT | 0.000 |
| 69 | L lateral occipital, CT | 0.057 | 144 | L parahippocampal, CT | 0.000 |
| 70 | L banks sts, SA | 0.054 | 145 | R pars orbitalis, CT | 0.000 |
| 71 | L parahippocampal, SA | 0.051 | 146 | L frontal pole, CT | 0.000 |
| 72 | R caudal anterior cingulate, SA | 0.051 | 147 | R rostral anterior cingulate, CT | 0.000 |
| 73 | R parahippocampal, SA | 0.050 | 148 | L pars triangularis, CT | 0.000 |
| 74 | R postcentral, CT | 0.050 | 149 | R entorhinal, CT | 0.000 |
| 75 | R entorhinal, SA | 0.045 | 150 | L temporal pole, CT | 0.000 |

**Note.** SA, surface area; CT, cortical thickness; sts, superior temporal sulcus.

**Table S7.** **Psychosis subtype comparisons on** **top 35 ranking model features contributing to participant similarity as determined by NMI.**

| No. | Top Contributing Features | NMI | Contrast | Cohen’s *d* | SE | 95% CI | *t*-value | *p*FDR |
| --- | --- | --- | --- | --- | --- | --- | --- | --- |
| 1 | R hippocampus | 0.258 | S1-HC | 0.057 | 0.09 | [-0.13,0.24] | 0.61 | 0.697 |
|  |  |  | **S2-HC** | **-0.929** | **0.11** | **[-1.15,-0.71]** | **-8.47** | **2.07e-15** |
| 2 | L amygdala | 0.219 | S1-HC | 0.128 | 0.09 | [-0.06,0.31] | 1.36 | 0.242 |
|  |  |  | **S2-HC** | **-0.887** | **0.11** | **[-1.11,-0.67]** | **-8.08** | **2.05e-14** |
| 3 | L hippocampus | 0.212 | S1-HC | -0.002 | 0.09 | [-0.19,0.18] | -0.03 | 0.979 |
|  |  |  | **S2-HC** | **-0.926** | **0.11** | **[-1.15,-0.7]** | **-8.44** | **2.07e-15** |
| 4 | R amygdala | 0.194 | S1-HC | -0.027 | 0.09 | [-0.21,0.16] | -0.29 | 0.927 |
|  |  |  | **S2-HC** | **-0.830** | **0.11** | **[-1.05,-0.61]** | **-7.57** | **6.35e-13** |
| 5 | L medial orbitofrontal, SA | 0.191 | **S1-HC** | **0.405** | **0.09** | **[0.22,0.59]** | **4.40** | **1.87e-05** |
|  |  |  | **S2-HC** | **-0.829** | **0.11** | **[-1.04,-0.62]** | **-7.90** | **8.28e-14** |
| 6 | R inferior temporal, SA | 0.188 | **S1-HC** | **0.346** | **0.09** | **[0.16,0.53]** | **3.76** | **2.32e-04** |
|  |  |  | **S2-HC** | **-0.919** | **0.11** | **[-1.13,-0.71]** | **-8.77** | **3.09e-16** |
| 7 | L precuneus, SA | 0.186 | **S1-HC** | **0.516** | **0.09** | **[0.33,0.7]** | **5.60** | **6.46e-08** |
|  |  |  | **S2-HC** | **-0.833** | **0.11** | **[-1.04,-0.62]** | **-7.95** | **6.88e-14** |
| 8 | R precuneus, SA | 0.185 | **S1-HC** | **0.492** | **0.09** | **[0.31,0.68]** | **5.34** | **2.54e-07** |
|  |  |  | **S2-HC** | **-0.866** | **0.11** | **[-1.08,-0.66]** | **-8.26** | **7.80e-15** |
| 9 | L precentral, SA | 0.177 | **S1-HC** | **0.332** | **0.09** | **[0.15,0.51]** | **3.60** | **3.95e-04** |
|  |  |  | **S2-HC** | **-0.739** | **0.11** | **[-0.95,-0.53]** | **-7.05** | **1.61e-11** |
| 10 | L thalamus | 0.174 | S1-HC | 0.003 | 0.09 | [-0.18,0.19] | 0.03 | 0.979 |
|  |  |  | **S2-HC** | **-0.793** | **0.11** | **[-1.01,-0.57]** | **-7.22** | **5.42e-12** |
| 11 | R fusiform, SA | 0.169 | **S1-HC** | **0.297** | **0.09** | **[0.12,0.48]** | **3.23** | **0.001** |
|  |  |  | **S2-HC** | **-1.003** | **0.11** | **[-1.22,-0.79]** | **-9.57** | **1.56e-18** |
| 12 | R thalamus | 0.166 | S1-HC | -0.021 | 0.09 | [-0.21,0.16] | -0.22 | 0.927 |
|  |  |  | **S2-HC** | **-0.770** | **0.11** | **[-0.99,-0.55]** | **-7.02** | **1.80e-11** |
| 13 | R middle temporal, SA | 0.163 | **S1-HC** | **0.3** | **0.09** | **[0.12,0.48]** | **3.26** | **0.001** |
|  |  |  | **S2-HC** | **-0.907** | **0.11** | **[-1.12,-0.7]** | **-8.65** | **5.76e-16** |
| 14 | L inferior temporal, SA | 0.160 | **S1-HC** | **0.233** | **0.09** | **[0.05,0.42]** | **2.53** | **0.012** |
|  |  |  | **S2-HC** | **-0.902** | **0.11** | **[-1.11,-0.69]** | **-8.60** | **6.84e-16** |
| 15 | L superior parietal, SA | 0.155 | **S1-HC** | **0.452** | **0.09** | **[0.27,0.64]** | **4.91** | **2.11e-06** |
|  |  |  | **S2-HC** | **-0.758** | **0.11** | **[-0.97,-0.55]** | **-7.23** | **4.99e-12** |
| 16 | L postcentral, SA | 0.152 | **S1-HC** | **0.345** | **0.09** | **[0.16,0.53]** | **3.75** | **2.34e-04** |
|  |  |  | **S2-HC** | **-0.783** | **0.11** | **[-0.99,-0.57]** | **-7.46** | **1.26e-12** |
| 17 | R posterior cingulate, SA | 0.152 | **S1-HC** | **0.373** | **0.09** | **[0.19,0.56]** | **4.04** | **7.68e-05** |
|  |  |  | **S2-HC** | **-0.686** | **0.11** | **[-0.9,-0.48]** | **-6.54** | **3.22e-10** |
| 18 | L caudate | 0.150 | **S1-HC** | **0.486** | **0.1** | **[0.3,0.67]** | **5.17** | **8.02e-07** |
|  |  |  | **S2-HC** | **-0.329** | **0.11** | **[-0.55,-0.11]** | **-3.00** | **0.005** |
| 19 | L rostral middle frontal, SA | 0.149 | **S1-HC** | **0.427** | **0.09** | **[0.24,0.61]** | **4.63** | **6.95e-06** |
|  |  |  | **S2-HC** | **-0.727** | **0.11** | **[-0.94,-0.52]** | **-6.93** | **3.02e-11** |
| 20 | R rostral middle frontal, SA | 0.149 | **S1-HC** | **0.425** | **0.09** | **[0.24,0.61]** | **4.62** | **7.31e-06** |
|  |  |  | **S2-HC** | **-0.68** | **0.11** | **[-0.89,-0.47]** | **-6.48** | **4.38e-10** |
| 21 | L middle temporal, SA | 0.145 | **S1-HC** | **0.215** | **0.09** | **[0.03,0.4]** | **2.33** | **0.02** |
|  |  |  | **S2-HC** | **-0.93** | **0.11** | **[-1.14,-0.72]** | **-8.87** | **2.02e-16** |
| 22 | L superior frontal, SA | 0.143 | **S1-HC** | **0.381** | **0.09** | **[0.2,0.56]** | **4.14** | **5.48e-05** |
|  |  |  | **S2-HC** | **-0.732** | **0.11** | **[-0.94,-0.52]** | **-6.98** | **2.37e-11** |
| 23 | L superior temporal, SA | 0.142 | **S1-HC** | **0.332** | **0.09** | **[0.15,0.51]** | **3.61** | **3.95e-04** |
|  |  |  | **S2-HC** | **-0.644** | **0.11** | **[-0.85,-0.44]** | **-6.14** | **3.00e-09** |
| 24 | R medial orbitofrontal, SA | 0.141 | **S1-HC** | **0.433** | **0.09** | **[0.25,0.62]** | **4.70** | **5.29e-06** |
|  |  |  | **S2-HC** | **-0.651** | **0.11** | **[-0.86,-0.44]** | **-6.21** | **2.10e-09** |
| 25 | R superior parietal, SA | 0.140 | **S1-HC** | **0.386** | **0.09** | **[0.2,0.57]** | **4.19** | **4.57e-05** |
|  |  |  | **S2-HC** | **-0.692** | **0.11** | **[-0.9,-0.48]** | **-6.60** | **2.30e-10** |
| 26 | R caudate | 0.139 | **S1-HC** | **0.44** | **0.1** | **[0.25,0.63]** | **4.69** | **7.70e-06** |
|  |  |  | **S2-HC** | **-0.336** | **0.11** | **[-0.55,-0.12]** | **-3.06** | **0.005** |
| 27 | L fusiform, SA | 0.139 | **S1-HC** | **0.295** | **0.09** | **[0.11,0.48]** | **3.20** | **0.001** |
|  |  |  | **S2-HC** | **-0.787** | **0.11** | **[-1,-0.58]** | **-7.50** | **1.03e-12** |
| 28 | R superior frontal, SA | 0.135 | **S1-HC** | **0.354** | **0.09** | **[0.17,0.54]** | **3.84** | **1.72e-04** |
|  |  |  | **S2-HC** | **-0.818** | **0.11** | **[-1.03,-0.61]** | **-7.80** | **1.52e-13** |
| 29 | R precentral, SA | 0.129 | **S1-HC** | **0.423** | **0.09** | **[0.24,0.61]** | **4.60** | **7.77e-06** |
|  |  |  | **S2-HC** | **-0.807** | **0.11** | **[-1.02,-0.6]** | **-7.70** | **2.84e-13** |
| 30 | L putamen | 0.126 | **S1-HC** | **0.24** | **0.09** | **[0.05,0.43]** | **2.55** | **0.016** |
|  |  |  | **S2-HC** | **-0.33** | **0.11** | **[-0.55,-0.11]** | **-3.01** | **0.005** |
| 31 | R postcentral, SA | 0.126 | **S1-HC** | **0.433** | **0.09** | **[0.25,0.62]** | **4.70** | **5.29e-06** |
|  |  |  | **S2-HC** | **-0.778** | **0.11** | **[-0.99,-0.57]** | **-7.42** | **1.61e-12** |
| 32 | R supramarginal, SA | 0.125 | **S1-HC** | **0.432** | **0.09** | **[0.25,0.61]** | **4.69** | **5.52e-06** |
|  |  |  | **S2-HC** | **-0.533** | **0.11** | **[-0.74,-0.33]** | **-5.08** | **9.12e-07** |
| 33 | L lateral orbitofrontal, SA | 0.122 | **S1-HC** | **0.378** | **0.09** | **[0.2,0.56]** | **4.10** | **6.17e-05** |
|  |  |  | **S2-HC** | **-0.714** | **0.11** | **[-0.92,-0.51]** | **-6.81** | **6.26e-11** |
| 34 | L pars orbitalis, SA | 0.119 | **S1-HC** | **0.31** | **0.09** | **[0.13,0.49]** | **3.37** | **8.82e-04** |
|  |  |  | **S2-HC** | **-0.762** | **0.11** | **[-0.97,-0.55]** | **-7.27** | **4.20e-12** |
| 35 | R superior temporal, SA | 0.116 | **S1-HC** | **0.314** | **0.09** | **[0.13,0.5]** | **3.41** | **7.71e-04** |
|  |  |  | **S2-HC** | **-0.666** | **0.11** | **[-0.88,-0.46]** | **-6.36** | **9.15e-10** |

**Note.** Data-driven psychosis subtype comparisons with healthy individuals using separate generalized linear models while including age, sex, scan version, and T1 image quality metric efc as covariates. Subcortical models additionally controlled for intracranial volume (ICV). FDR corrected using Benjamini-Hochberg procedure, regions with *p*_FDR_<0.05 are depicted in bold. S1, subtype 1; S2, subtype 2; HC, healthy control; SA, surface area; R, right; L, left.

**Table S8a.** **Cortical thickness comparisons among data-driven subtype 1 and healthy controls.**

| Region | Cohen’s *d*  (S1 v. HC) | SE | 95% CI | Estimate | *t* value | *p*-value | FDR  *p*-value |
| --- | --- | --- | --- | --- | --- | --- | --- |
| L banks STS | -0.090 | 0.092 | [-0.27,0.09] | -0.013 | -0.98 | 0.3272 | 0.5070 |
| R banks STS | -0.190 | 0.092 | [-0.37,-0.01] | -0.029 | -2.06 | 0.0400 | 0.1699 |
| L caudal anterior cingulate | -0.045 | 0.092 | [-0.23,0.14] | -0.008 | -0.49 | 0.6245 | 0.7864 |
| R caudal anterior cingulate | -0.150 | 0.092 | [-0.33,0.03] | -0.025 | -1.62 | 0.1048 | 0.2639 |
| L caudal middle frontal | -0.157 | 0.092 | [-0.34,0.02] | -0.021 | -1.70 | 0.0891 | 0.2396 |
| R caudal middle frontal | -0.058 | 0.092 | [-0.24,0.12] | -0.008 | -0.63 | 0.5285 | 0.7047 |
| L cuneus | 0.096 | 0.092 | [-0.09,0.28] | 0.013 | 1.04 | 0.2981 | 0.4826 |
| R cuneus | 0.156 | 0.092 | [-0.03,0.34] | 0.019 | 1.69 | 0.0916 | 0.2396 |
| L entorhinal | 0.100 | 0.092 | [-0.08,0.28] | 0.031 | 1.09 | 0.2764 | 0.4698 |
| R entorhinal | -0.157 | 0.092 | [-0.34,0.02] | -0.050 | -1.70 | 0.0895 | 0.2396 |
| L fusiform | -0.117 | 0.092 | [-0.3,0.06] | -0.013 | -1.27 | 0.2039 | 0.4078 |
| R fusiform | -0.107 | 0.092 | [-0.29,0.07] | -0.014 | -1.16 | 0.2475 | 0.4435 |
| L inferior parietal | -0.083 | 0.092 | [-0.26,0.1] | -0.010 | -0.90 | 0.3660 | 0.5410 |
| R inferior parietal | -0.187 | 0.092 | [-0.37,-0.01] | -0.022 | -2.03 | 0.0425 | 0.1699 |
| L inferior temporal | -0.063 | 0.092 | [-0.24,0.12] | -0.008 | -0.68 | 0.4946 | 0.6780 |
| R inferior temporal | -0.124 | 0.092 | [-0.31,0.06] | -0.017 | -1.35 | 0.1783 | 0.3910 |
| L isthmus cingulate | 0.041 | 0.092 | [-0.14,0.22] | 0.006 | 0.45 | 0.6537 | 0.8070 |
| R isthmus cingulate | -0.022 | 0.092 | [-0.2,0.16] | -0.003 | -0.24 | 0.8125 | 0.9057 |
| L lateral occipital | -0.089 | 0.092 | [-0.27,0.09] | -0.010 | -0.96 | 0.3355 | 0.5070 |
| R lateral occipital | -0.090 | 0.092 | [-0.27,0.09] | -0.010 | -0.97 | 0.3305 | 0.5070 |
| L lateral orbitofrontal | -0.111 | 0.092 | [-0.29,0.07] | -0.016 | -1.20 | 0.2297 | 0.4435 |
| R lateral orbitofrontal | -0.139 | 0.092 | [-0.32,0.04] | -0.017 | -1.51 | 0.1321 | 0.3097 |
| L lingual | 0.006 | 0.092 | [-0.18,0.19] | 0.001 | 0.07 | 0.9456 | 0.9598 |
| R lingual | -0.012 | 0.092 | [-0.19,0.17] | -0.001 | -0.13 | 0.8964 | 0.9378 |
| L medial orbitofrontal | -0.143 | 0.092 | [-0.32,0.04] | -0.018 | -1.55 | 0.1225 | 0.2975 |
| R medial orbitofrontal | -0.122 | 0.092 | [-0.3,0.06] | -0.014 | -1.32 | 0.1865 | 0.3963 |
| L middle temporal | -0.180 | 0.092 | [-0.36,0.002] | -0.025 | -1.95 | 0.0518 | 0.1826 |
| R middle temporal | -0.201 | 0.092 | [-0.38,-0.02] | -0.027 | -2.18 | 0.0294 | 0.1335 |
| L parahippocampal | -0.157 | 0.092 | [-0.34,0.02] | -0.039 | -1.70 | 0.0891 | 0.2396 |
| R parahippocampal | -0.109 | 0.092 | [-0.29,0.07] | -0.022 | -1.18 | 0.2373 | 0.4435 |
| L paracentral | -0.062 | 0.092 | [-0.24,0.12] | -0.009 | -0.68 | 0.4985 | 0.6780 |
| R paracentral | -0.036 | 0.092 | [-0.22,0.15] | -0.005 | -0.39 | 0.6960 | 0.8070 |
| **L pars opercularis** | **-0.329** | **0.093** | **[-0.51,-0.15]** | **-0.049** | **-3.58** | **0.0004** | **0.0256** |
| R pars opercularis | -0.036 | 0.092 | [-0.22,0.15] | -0.005 | -0.39 | 0.7002 | 0.8070 |
| L pars orbitalis | -0.247 | 0.092 | [-0.43,-0.07] | -0.046 | -2.68 | 0.0076 | 0.0688 |
| R pars orbitalis | -0.239 | 0.092 | [-0.42,-0.06] | -0.045 | -2.59 | 0.0097 | 0.0688 |
| L pars triangularis | -0.288 | 0.093 | [-0.47,-0.11] | -0.043 | -3.13 | 0.0019 | 0.0631 |
| R pars triangularis | -0.236 | 0.092 | [-0.42,-0.05] | -0.036 | -2.56 | 0.0107 | 0.0688 |
| L pericalcarine | 0.099 | 0.092 | [-0.08,0.28] | 0.014 | 1.07 | 0.2852 | 0.4730 |
| R pericalcarine | 0.178 | 0.092 | [-0.003,0.36] | 0.023 | 1.93 | 0.0537 | 0.1826 |
| L postcentral | -0.016 | 0.092 | [-0.2,0.17] | -0.002 | -0.17 | 0.8645 | 0.9378 |
| R postcentral | 0.036 | 0.092 | [-0.15,0.22] | 0.004 | 0.39 | 0.6986 | 0.8070 |
| L posterior cingulate | 0.013 | 0.092 | [-0.17,0.19] | 0.002 | 0.14 | 0.8895 | 0.9378 |
| R posterior cingulate | -0.168 | 0.092 | [-0.35,0.01] | -0.021 | -1.82 | 0.0689 | 0.2232 |
| L precentral | -0.248 | 0.092 | [-0.43,-0.07] | -0.032 | -2.69 | 0.0074 | 0.0688 |
| R precentral | -0.107 | 0.092 | [-0.29,0.07] | -0.013 | -1.16 | 0.2478 | 0.4435 |
| L precuneus | -0.046 | 0.092 | [-0.23,0.14] | -0.005 | -0.50 | 0.6170 | 0.7864 |
| R precuneus | -0.074 | 0.092 | [-0.26,0.11] | -0.009 | -0.81 | 0.4197 | 0.6073 |
| L rostral anterior cingulate | -0.135 | 0.092 | [-0.32,0.05] | -0.024 | -1.47 | 0.1423 | 0.3227 |
| R rostral anterior cingulate | 0.007 | 0.092 | [-0.17,0.19] | 0.001 | 0.08 | 0.9372 | 0.9598 |
| L rostral middle frontal | -0.255 | 0.092 | [-0.44,-0.07] | -0.030 | -2.77 | 0.0058 | 0.0688 |
| R rostral middle frontal | -0.254 | 0.092 | [-0.44,-0.07] | -0.031 | -2.75 | 0.0061 | 0.0688 |
| L superior frontal | -0.256 | 0.092 | [-0.44,-0.07] | -0.035 | -2.78 | 0.0056 | 0.0688 |
| R superior frontal | -0.221 | 0.092 | [-0.4,-0.04] | -0.031 | -2.40 | 0.0166 | 0.0866 |
| L superior parietal | 0.063 | 0.092 | [-0.12,0.24] | 0.007 | 0.69 | 0.4928 | 0.6780 |
| R superior parietal | 0.013 | 0.092 | [-0.17,0.19] | 0.001 | 0.14 | 0.8917 | 0.9378 |
| L superior temporal | -0.183 | 0.092 | [-0.36,-0.002] | -0.027 | -1.99 | 0.0471 | 0.1780 |
| R superior temporal | -0.235 | 0.092 | [-0.42,-0.05] | -0.034 | -2.55 | 0.0111 | 0.0688 |
| L supramarginal | -0.213 | 0.092 | [-0.4,-0.03] | -0.027 | -2.32 | 0.0209 | 0.1013 |
| R supramarginal | -0.120 | 0.092 | [-0.3,0.06] | -0.015 | -1.30 | 0.1949 | 0.4017 |
| L frontal pole | -0.225 | 0.092 | [-0.41,-0.04] | -0.056 | -2.45 | 0.0147 | 0.0832 |
| R frontal pole | 0.034 | 0.092 | [-0.15,0.22] | 0.007 | 0.37 | 0.7132 | 0.8083 |
| L temporal pole | 0.004 | 0.092 | [-0.18,0.19] | 0.001 | 0.05 | 0.9625 | 0.9625 |
| R temporal pole | -0.038 | 0.092 | [-0.22,0.14] | -0.012 | -0.42 | 0.6778 | 0.8070 |
| L transverse temporal | -0.162 | 0.092 | [-0.34,0.02] | -0.032 | -1.76 | 0.0793 | 0.2396 |
| R transverse temporal | -0.242 | 0.092 | [-0.42,-0.06] | -0.048 | -2.62 | 0.0089 | 0.0688 |
| L insula | -0.104 | 0.092 | [-0.29,0.08] | -0.016 | -1.13 | 0.2587 | 0.4510 |
| R insula | -0.055 | 0.092 | [-0.24,0.13] | -0.009 | -0.60 | 0.5504 | 0.7198 |

**Note.** General linear models per region controlling for age, sex, scan version, and T1w quality (MRIQC efc). FDR corrected using Benjamini-Hochberg procedure across all CT regions. Regions with *p*_FDR_<0.05 are depicted in bold. S1 = subtype 1, HC = healthy control, SE = standard error, L = left, R = right.

**Table S8b.** **Cortical thickness comparisons among data-driven subtype 2 and healthy controls.**

| Region | Cohen’s *d*  (S2 v. HC) | SE | 95% CI | Estimate | *t* value | *p*-value | FDR  *p*-value |
| --- | --- | --- | --- | --- | --- | --- | --- |
| **L banks STS** | **-0.633** | **0.106** | **[-0.84,-0.42]** | **-0.093** | **-6.03** | **2.778e-09** | **2.1004e-08** |
| **R banks STS** | **-0.590** | **0.106** | **[-0.8,-0.38]** | **-0.090** | **-5.63** | **2.765e-08** | **1.4463e-07** |
| **L caudal anterior cingulate** | **-0.293** | **0.105** | **[-0.5,-0.09]** | **-0.053** | **-2.79** | **5.450e-03** | **7.2665e-03** |
| **R caudal anterior cingulate** | **-0.230** | **0.105** | **[-0.44,-0.02]** | **-0.038** | **-2.20** | **0.0285** | **0.0346** |
| **L caudal middle frontal** | **-0.462** | **0.106** | **[-0.67,-0.25]** | **-0.062** | **-4.41** | **1.237e-05** | **2.5496e-05** |
| **R caudal middle frontal** | **-0.513** | **0.106** | **[-0.72,-0.31]** | **-0.067** | **-4.89** | **1.301e-06** | **4.0217e-06** |
| **L cuneus** | **-0.241** | **0.105** | **[-0.45,-0.03]** | **-0.033** | **-2.29** | **0.0221** | **0.0274** |
| **R cuneus** | **-0.273** | **0.105** | **[-0.48,-0.07]** | **-0.033** | **-2.60** | **9.424e-03** | **0.0121** |
| L entorhinal | -0.126 | 0.105 | [-0.33,0.08] | -0.039 | -1.20 | 0.2311 | 0.2455 |
| R entorhinal | -0.174 | 0.105 | [-0.38,0.03] | -0.056 | -1.66 | 0.0967 | 0.1079 |
| **L fusiform** | **-0.667** | **0.107** | **[-0.88,-0.46]** | **-0.077** | **-6.36** | **3.984e-10** | **9.0667e-09** |
| **R fusiform** | **-0.519** | **0.106** | **[-0.73,-0.31]** | **-0.070** | **-4.95** | **9.681e-07** | **3.2917e-06** |
| **L inferior parietal** | **-0.499** | **0.106** | **[-0.71,-0.29]** | **-0.061** | **-4.76** | **2.404e-06** | **6.5402e-06** |
| **R inferior parietal** | **-0.596** | **0.106** | **[-0.81,-0.39]** | **-0.070** | **-5.69** | **2.017e-08** | **1.1430e-07** |
| **L inferior temporal** | **-0.298** | **0.105** | **[-0.5,-0.09]** | **-0.038** | **-2.84** | **4.677e-03** | **6.3605e-03** |
| **R inferior temporal** | **-0.424** | **0.106** | **[-0.63,-0.22]** | **-0.060** | **-4.04** | **6.026e-05** | **1.2053e-04** |
| L isthmus cingulate | -0.196 | 0.105 | [-0.4,0.01] | -0.031 | -1.87 | 0.0622 | 0.0717 |
| R isthmus cingulate | -0.179 | 0.105 | [-0.38,0.03] | -0.028 | -1.70 | 0.0888 | 0.1006 |
| **L lateral occipital** | **-0.633** | **0.106** | **[-0.84,-0.42]** | **-0.068** | **-6.04** | **2.675e-09** | **2.1004e-08** |
| **R lateral occipital** | **-0.639** | **0.106** | **[-0.85,-0.43]** | **-0.070** | **-6.09** | **1.978e-09** | **1.9234e-08** |
| **L lateral orbitofrontal** | **-0.278** | **0.105** | **[-0.48,-0.07]** | **-0.040** | **-2.65** | **8.300e-03** | **0.0109** |
| **R lateral orbitofrontal** | **-0.339** | **0.105** | **[-0.55,-0.13]** | **-0.040** | **-3.24** | **1.274e-03** | **1.9696e-03** |
| **L lingual** | **-0.388** | **0.106** | **[-0.6,-0.18]** | **-0.045** | **-3.70** | **2.339e-04** | **4.0781e-04** |
| **R lingual** | **-0.370** | **0.105** | **[-0.58,-0.16]** | **-0.041** | **-3.53** | **4.461e-04** | **7.5308e-04** |
| L medial orbitofrontal | -0.120 | 0.105 | [-0.33,0.09] | -0.015 | -1.15 | 0.2518 | 0.2635 |
| R medial orbitofrontal | -0.146 | 0.105 | [-0.35,0.06] | -0.017 | -1.40 | 0.1633 | 0.1787 |
| **L middle temporal** | **-0.517** | **0.106** | **[-0.73,-0.31]** | **-0.071** | **-4.93** | **1.036e-06** | **3.3555e-06** |
| **R middle temporal** | **-0.585** | **0.106** | **[-0.79,-0.38]** | **-0.079** | **-5.57** | **3.727e-08** | **1.8103e-07** |
| **L parahippocampal** | **-0.402** | **0.106** | **[-0.61,-0.2]** | **-0.100** | **-3.84** | **1.382e-04** | **2.4734e-04** |
| **R parahippocampal** | **-0.304** | **0.105** | **[-0.51,-0.1]** | **-0.063** | **-2.90** | **3.902e-03** | **5.5280e-03** |
| **L paracentral** | **-0.527** | **0.106** | **[-0.74,-0.32]** | **-0.073** | **-5.02** | **6.712e-07** | **2.4023e-06** |
| **R paracentral** | **-0.408** | **0.106** | **[-0.62,-0.2]** | **-0.057** | **-3.89** | **1.120e-04** | **2.0588e-04** |
| **L pars opercularis** | **-0.554** | **0.106** | **[-0.76,-0.35]** | **-0.082** | **-5.28** | **1.810e-07** | **7.6934e-07** |
| **R pars opercularis** | **-0.529** | **0.106** | **[-0.74,-0.32]** | **-0.079** | **-5.05** | **5.965e-07** | **2.2536e-06** |
| **L pars orbitalis** | **-0.244** | **0.105** | **[-0.45,-0.04]** | **-0.045** | **-2.32** | **0.0205** | **0.0258** |
| **R pars orbitalis** | **-0.310** | **0.105** | **[-0.52,-0.1]** | **-0.058** | **-2.96** | **3.210e-03** | **4.7162e-03** |
| **L pars triangularis** | **-0.414** | **0.106** | **[-0.62,-0.21]** | **-0.062** | **-3.95** | **8.773e-05** | **1.6571e-04** |
| **R pars triangularis** | **-0.501** | **0.106** | **[-0.71,-0.29]** | **-0.077** | **-4.78** | **2.194e-06** | **6.2171e-06** |
| L pericalcarine | -0.028 | 0.105 | [-0.23,0.18] | -0.004 | -0.26 | 0.7931 | 0.7931 |
| R pericalcarine | -0.207 | 0.105 | [-0.41,-0.0005] | -0.027 | -1.97 | 0.0491 | 0.0576 |
| **L postcentral** | **-0.550** | **0.106** | **[-0.76,-0.34]** | **-0.059** | **-5.24** | **2.188e-07** | **8.7532e-07** |
| **R postcentral** | **-0.655** | **0.107** | **[-0.87,-0.45]** | **-0.070** | **-6.25** | **7.678e-10** | **1.0608e-08** |
| **L posterior cingulate** | **-0.370** | **0.105** | **[-0.58,-0.16]** | **-0.049** | **-3.53** | **4.541e-04** | **7.5308e-04** |
| **R posterior cingulate** | **-0.310** | **0.105** | **[-0.52,-0.1]** | **-0.038** | **-2.95** | **3.260e-03** | **4.7162e-03** |
| **L precentral** | **-0.655** | **0.107** | **[-0.86,-0.45]** | **-0.084** | **-6.25** | **7.799e-10** | **1.0608e-08** |
| **R precentral** | **-0.688** | **0.107** | **[-0.9,-0.48]** | **-0.086** | **-6.56** | **1.113e-10** | **5.7800e-09** |
| **L precuneus** | **-0.498** | **0.106** | **[-0.71,-0.29]** | **-0.058** | **-4.75** | **2.587e-06** | **6.7656e-06** |
| **R precuneus** | **-0.464** | **0.106** | **[-0.67,-0.26]** | **-0.055** | **-4.42** | **1.159e-05** | **2.4631e-05** |
| **L rostral anterior cingulate** | **-0.300** | **0.105** | **[-0.51,-0.09]** | **-0.053** | **-2.86** | **4.384e-03** | **6.0836e-03** |
| R rostral anterior cingulate | 0.103 | 0.105 | [-0.1,0.31] | 0.019 | 0.99 | 0.3243 | 0.3342 |
| **L rostral middle frontal** | **-0.418** | **0.106** | **[-0.63,-0.21]** | **-0.049** | **-3.98** | **7.568e-05** | **1.4704e-04** |
| **R rostral middle frontal** | **-0.464** | **0.106** | **[-0.67,-0.26]** | **-0.056** | **-4.43** | **1.121e-05** | **2.4586e-05** |
| **L superior frontal** | **-0.482** | **0.106** | **[-0.69,-0.27]** | **-0.065** | **-4.59** | **5.265e-06** | **1.2786e-05** |
| **R superior frontal** | **-0.505** | **0.106** | **[-0.71,-0.3]** | **-0.071** | **-4.82** | **1.811e-06** | **5.3541e-06** |
| **L superior parietal** | **-0.479** | **0.106** | **[-0.69,-0.27]** | **-0.056** | **-4.57** | **5.901e-06** | **1.3377e-05** |
| **R superior parietal** | **-0.489** | **0.106** | **[-0.7,-0.28]** | **-0.056** | **-4.66** | **3.806e-06** | **9.5843e-06** |
| **L superior temporal** | **-0.604** | **0.106** | **[-0.81,-0.4]** | **-0.091** | **-5.76** | **1.318e-08** | **8.1476e-08** |
| **R superior temporal** | **-0.682** | **0.107** | **[-0.89,-0.47]** | **-0.100** | **-6.50** | **1.673e-10** | **5.7800e-09** |
| **L supramarginal** | **-0.572** | **0.106** | **[-0.78,-0.36]** | **-0.073** | **-5.45** | **7.143e-08** | **3.2382e-07** |
| **R supramarginal** | **-0.640** | **0.106** | **[-0.85,-0.43]** | **-0.079** | **-6.10** | **1.886e-09** | **1.9234e-08** |
| **L frontal pole** | **-0.354** | **0.105** | **[-0.56,-0.15]** | **-0.089** | **-3.37** | **7.917e-04** | **1.2819e-03** |
| R frontal pole | -0.081 | 0.105 | [-0.29,0.13] | -0.017 | -0.78 | 0.4378 | 0.4443 |
| L temporal pole | -0.208 | 0.105 | [-0.41,-0.002] | -0.064 | -1.99 | 0.0475 | 0.0567 |
| R temporal pole | -0.146 | 0.105 | [-0.35,0.06] | -0.047 | -1.39 | 0.1655 | 0.1787 |
| **L transverse temporal** | **-0.480** | **0.106** | **[-0.69,-0.27]** | **-0.094** | **-4.58** | **5.700e-06** | **1.3366e-05** |
| **R transverse temporal** | **-0.617** | **0.106** | **[-0.83,-0.41]** | **-0.123** | **-5.88** | **6.750e-09** | **4.5900e-08** |
| **L insula** | **-0.346** | **0.105** | **[-0.55,-0.14]** | **-0.054** | **-3.30** | **1.011e-03** | **1.5989e-03** |
| **R insula** | **-0.327** | **0.105** | **[-0.53,-0.12]** | **-0.051** | **-3.12** | **1.921e-03** | **2.9025e-03** |

**Note.** General linear models per region controlling for age, sex, scan version, and T1w quality (MRIQC efc). FDR corrected using Benjamini-Hochberg procedure across all CT regions. Regions with *p*_FDR_<0.05 are depicted in bold. S2 = subtype 2, HC = healthy control, SE = standard error, L = left, R = right.

**Table S9a.** **Surface area comparisons among data-driven subtype 1 and healthy controls.**

| Region | Cohen’s *d*  (S1 v. HC) | SE | 95% CI | Estimate | *t* value | *p*-value | FDR  *p*-value |
| --- | --- | --- | --- | --- | --- | --- | --- |
| L banks STS | 0.122 | 0.092 | [-0.06,0.3] | 19.70 | 1.32 | 0.1866 | 0.1923 |
| **R banks STS** | **0.209** | **0.092** | **[0.03,0.39]** | **26.14** | **2.27** | **0.0236** | **0.0282** |
| **L caudal anterior cingulate** | **0.207** | **0.092** | **[0.03,0.39]** | **26.84** | **2.25** | **0.0249** | **0.0292** |
| R caudal anterior cingulate | 0.152 | 0.092 | [-0.03,0.33] | 22.30 | 1.65 | 0.0990 | 0.1068 |
| **L caudal middle frontal** | **0.257** | **0.092** | **[0.08,0.44]** | **86.56** | **2.79** | **5.4882e-03** | **7.6163e-03** |
| **R caudal middle frontal** | **0.271** | **0.092** | **[0.09,0.45]** | **92.09** | **2.94** | **3.3679e-03** | **5.2050e-03** |
| **L cuneus** | **0.364** | **0.093** | **[0.18,0.55]** | **72.48** | **3.95** | **8.8491e-05** | **3.3430e-04** |
| **R cuneus** | **0.423** | **0.093** | **[0.24,0.61]** | **83.53** | **4.59** | **5.3017e-06** | **3.6054e-05** |
| L entorhinal | 0.099 | 0.092 | [-0.08,0.28] | 8.82 | 1.07 | 0.2856 | 0.2898 |
| R entorhinal | 0.122 | 0.092 | [-0.06,0.3] | 9.64 | 1.33 | 0.1847 | 0.1923 |
| **L fusiform** | **0.295** | **0.093** | **[0.11,0.48]** | **97.90** | **3.20** | **1.4270e-03** | **2.6227e-03** |
| **R fusiform** | **0.297** | **0.093** | **[0.12,0.48]** | **86.89** | **3.23** | **1.3223e-03** | **2.5690e-03** |
| **L inferior parietal** | **0.229** | **0.092** | **[0.05,0.41]** | **128.78** | **2.49** | **0.0132** | **0.0170** |
| R inferior parietal | 0.185 | 0.092 | [0.004,0.37] | 123.34 | 2.01 | 0.0452 | 0.0513 |
| **L inferior temporal** | **0.233** | **0.092** | **[0.05,0.42]** | **101.11** | **2.53** | **0.0116** | **0.0151** |
| **R inferior temporal** | **0.346** | **0.093** | **[0.16,0.53]** | **131.99** | **3.76** | **1.8754e-04** | **5.9737e-04** |
| **L isthmus cingulate** | **0.414** | **0.093** | **[0.23,0.6]** | **58.97** | **4.49** | **8.5431e-06** | **5.2811e-05** |
| **R isthmus cingulate** | **0.354** | **0.093** | **[0.17,0.54]** | **44.13** | **3.84** | **1.3427e-04** | **4.6193e-04** |
| **L lateral occipital** | **0.382** | **0.093** | **[0.2,0.56]** | **214.50** | **4.14** | **3.9285e-05** | **1.8137e-04** |
| **R lateral occipital** | **0.285** | **0.093** | **[0.1,0.47]** | **162.56** | **3.10** | **2.0455e-03** | **3.4773e-03** |
| **L lateral orbitofrontal** | **0.378** | **0.093** | **[0.2,0.56]** | **107.00** | **4.10** | **4.6249e-05** | **1.9656e-04** |
| **R lateral orbitofrontal** | **0.275** | **0.093** | **[0.09,0.46]** | **88.01** | **2.99** | **2.9353e-03** | **4.7523e-03** |
| **L lingual** | **0.264** | **0.092** | **[0.08,0.45]** | **99.60** | **2.87** | **4.2581e-03** | **6.1017e-03** |
| **R lingual** | **0.194** | **0.092** | **[0.01,0.38]** | **77.03** | **2.10** | **0.0359** | **0.0413** |
| **L medial orbitofrontal** | **0.405** | **0.093** | **[0.22,0.59]** | **80.62** | **4.40** | **1.2941e-05** | **7.3332e-05** |
| **R medial orbitofrontal** | **0.433** | **0.093** | **[0.25,0.62]** | **86.99** | **4.70** | **3.1559e-06** | **3.6054e-05** |
| **L middle temporal** | **0.215** | **0.092** | **[0.03,0.4]** | **80.55** | **2.33** | **0.0201** | **0.0248** |
| **R middle temporal** | **0.300** | **0.093** | **[0.12,0.48]** | **119.93** | **3.26** | **1.1809e-03** | **2.3618e-03** |
| L parahippocampal | 0.170 | 0.092 | [-0.01,0.35] | 11.89 | 1.85 | 0.0651 | 0.0725 |
| **R parahippocampal** | **0.245** | **0.092** | **[0.06,0.43]** | **18.06** | **2.66** | **8.0737e-03** | **0.0108** |
| **L paracentral** | **0.296** | **0.093** | **[0.12,0.48]** | **45.51** | **3.22** | **1.3669e-03** | **2.5819e-03** |
| **R paracentral** | **0.323** | **0.093** | **[0.14,0.51]** | **54.05** | **3.51** | **4.7885e-04** | **1.2524e-03** |
| **L pars opercularis** | **0.301** | **0.093** | **[0.12,0.48]** | **73.88** | **3.27** | **1.1460e-03** | **2.3615e-03** |
| **R pars opercularis** | **0.276** | **0.093** | **[0.09,0.46]** | **52.63** | **2.99** | **2.8670e-03** | **4.7523e-03** |
| **L pars orbitalis** | **0.310** | **0.093** | **[0.13,0.49]** | **27.13** | **3.37** | **7.9711e-04** | **1.7485e-03** |
| **R pars orbitalis** | **0.319** | **0.093** | **[0.14,0.5]** | **33.75** | **3.46** | **5.6907e-04** | **1.4332e-03** |
| **L pars triangularis** | **0.250** | **0.092** | **[0.07,0.43]** | **47.28** | **2.72** | **6.7942e-03** | **9.2401e-03** |
| **R pars triangularis** | **0.270** | **0.092** | **[0.09,0.45]** | **59.37** | **2.94** | **3.4489e-03** | **5.2117e-03** |
| **L pericalcarine** | **0.314** | **0.093** | **[0.13,0.5]** | **77.16** | **3.41** | **6.9244e-04** | **1.6237e-03** |
| **R pericalcarine** | **0.264** | **0.092** | **[0.08,0.45]** | **65.73** | **2.87** | **4.3071e-03** | **6.1017e-03** |
| **L postcentral** | **0.345** | **0.093** | **[0.16,0.53]** | **144.58** | **3.75** | **1.9327e-04** | **5.9737e-04** |
| **R postcentral** | **0.433** | **0.093** | **[0.25,0.62]** | **172.38** | **4.70** | **3.1466e-06** | **3.6054e-05** |
| **L posterior cingulate** | **0.216** | **0.092** | **[0.03,0.4]** | **33.63** | **2.34** | **0.0196** | **0.0247** |
| **R posterior cingulate** | **0.373** | **0.093** | **[0.19,0.56]** | **58.02** | **4.04** | **5.9112e-05** | **2.3645e-04** |
| **L precentral** | **0.332** | **0.093** | **[0.15,0.51]** | **158.06** | **3.60** | **3.4223e-04** | **9.6965e-04** |
| **R precentral** | **0.423** | **0.093** | **[0.24,0.61]** | **181.11** | **4.60** | **5.2297e-06** | **3.6054e-05** |
| **L precuneus** | **0.516** | **0.093** | **[0.33,0.7]** | **213.71** | **5.60** | **3.2319e-08** | **2.1760e-06** |
| **R precuneus** | **0.492** | **0.093** | **[0.31,0.68]** | **212.49** | **5.34** | **1.3202e-07** | **4.4880e-06** |
| **L rostral anterior cingulate** | **0.307** | **0.093** | **[0.13,0.49]** | **47.62** | **3.34** | **9.0119e-04** | **1.9150e-03** |
| R rostral anterior cingulate | 0.083 | 0.092 | [-0.1,0.26] | 10.10 | 0.90 | 0.3689 | 0.3689 |
| **L rostral middle frontal** | **0.427** | **0.093** | **[0.24,0.61]** | **270.07** | **4.63** | **4.4134e-06** | **3.6054e-05** |
| **R rostral middle frontal** | **0.425** | **0.093** | **[0.24,0.61]** | **294.32** | **4.62** | **4.7817e-06** | **3.6054e-05** |
| **L superior frontal** | **0.381** | **0.093** | **[0.2,0.56]** | **289.68** | **4.14** | **4.0007e-05** | **1.8137e-04** |
| **R superior frontal** | **0.354** | **0.093** | **[0.17,0.54]** | **265.48** | **3.84** | **1.3586e-04** | **4.6193e-04** |
| **L superior parietal** | **0.452** | **0.093** | **[0.27,0.64]** | **281.31** | **4.91** | **1.1771e-06** | **2.6679e-05** |
| **R superior parietal** | **0.386** | **0.093** | **[0.2,0.57]** | **222.31** | **4.19** | **3.2532e-05** | **1.7017e-04** |
| **L superior temporal** | **0.332** | **0.093** | **[0.15,0.51]** | **138.96** | **3.61** | **3.3400e-04** | **9.6965e-04** |
| **R superior temporal** | **0.314** | **0.093** | **[0.13,0.5]** | **114.41** | **3.41** | **6.8243e-04** | **1.6237e-03** |
| **L supramarginal** | **0.326** | **0.093** | **[0.14,0.51]** | **196.61** | **3.54** | **4.3549e-04** | **1.1845e-03** |
| **R supramarginal** | **0.432** | **0.093** | **[0.25,0.61]** | **200.25** | **4.69** | **3.3997e-06** | **3.6054e-05** |
| **L frontal pole** | **0.311** | **0.093** | **[0.13,0.49]** | **10.53** | **3.37** | **7.8961e-04** | **1.7485e-03** |
| **R frontal pole** | **0.265** | **0.092** | **[0.08,0.45]** | **11.47** | **2.88** | **4.1046e-03** | **6.0676e-03** |
| **L temporal pole** | **0.211** | **0.092** | **[0.03,0.39]** | **14.09** | **2.29** | **0.0225** | **0.0273** |
| R temporal pole | 0.159 | 0.092 | [-0.02,0.34] | 10.83 | 1.73 | 0.0845 | 0.0927 |
| **L transverse temporal** | **0.288** | **0.093** | **[0.11,0.47]** | **17.97** | **3.13** | **1.8204e-03** | **3.1740e-03** |
| **R transverse temporal** | **0.291** | **0.093** | **[0.11,0.47]** | **12.73** | **3.16** | **1.6596e-03** | **2.9698e-03** |
| L insula | 0.125 | 0.092 | [-0.06,0.31] | 34.15 | 1.36 | 0.1741 | 0.1849 |
| **R insula** | **0.272** | **0.092** | **[0.09,0.45]** | **59.40** | **2.96** | **3.2239e-03** | **5.0983e-03** |

**Note.** General linear models per region controlling for age, sex, scan version, and T1w quality (MRIQC efc). FDR corrected using Benjamini-Hochberg procedure across all SA regions. Regions with *p*_FDR_<0.05 are depicted in bold. S1 = subtype 1, HC = healthy control, SE = standard error, L = left, R = right.

**Table S9b. Surface area comparisons among data-driven subtype 2 and healthy controls.**

| Region | Cohen’s *d*  (S2 v. HC) | SE | 95% CI | Estimate | *t* value | *p*-value | FDR  *p*-value |
| --- | --- | --- | --- | --- | --- | --- | --- |
| **L banks STS** | **-0.476** | **0.106** | **[-0.68,-0.27]** | **-77.04** | **-4.54** | **6.7392e-06** | **8.6465e-06** |
| **R banks STS** | **-0.491** | **0.106** | **[-0.7,-0.28]** | **-61.39** | **-4.68** | **3.5292e-06** | **4.7997e-06** |
| **L caudal anterior cingulate** | **-0.422** | **0.106** | **[-0.63,-0.22]** | **-54.74** | **-4.03** | **6.3264e-05** | **7.0524e-05** |
| **R caudal anterior cingulate** | **-0.485** | **0.106** | **[-0.69,-0.28]** | **-71.09** | **-4.63** | **4.5178e-06** | **6.0238e-06** |
| **L caudal middle frontal** | **-0.577** | **0.106** | **[-0.79,-0.37]** | **-194.49** | **-5.50** | **5.5622e-08** | **9.0055e-08** |
| **R caudal middle frontal** | **-0.479** | **0.106** | **[-0.69,-0.27]** | **-162.75** | **-4.57** | **5.9107e-06** | **7.7293e-06** |
| **L cuneus** | **-0.615** | **0.106** | **[-0.82,-0.41]** | **-122.55** | **-5.86** | **7.4979e-09** | **1.3925e-08** |
| **R cuneus** | **-0.531** | **0.106** | **[-0.74,-0.32]** | **-104.74** | **-5.06** | **5.5616e-07** | **8.0466e-07** |
| **L entorhinal** | **-0.425** | **0.106** | **[-0.63,-0.22]** | **-38.07** | **-4.05** | **5.7027e-05** | **6.4631e-05** |
| **R entorhinal** | **-0.502** | **0.106** | **[-0.71,-0.29]** | **-39.58** | **-4.79** | **2.1096e-06** | **2.9886e-06** |
| **L fusiform** | **-0.787** | **0.107** | **[-1,-0.58]** | **-261.05** | **-7.50** | **2.1747e-13** | **1.3444e-12** |
| **R fusiform** | **-1.003** | **0.109** | **[-1.22,-0.79]** | **-293.40** | **-9.57** | **2.5895e-20** | **1.7680e-18** |
| **L inferior parietal** | **-0.691** | **0.107** | **[-0.9,-0.48]** | **-388.78** | **-6.59** | **9.4184e-11** | **2.5618e-10** |
| **R inferior parietal** | **-0.726** | **0.107** | **[-0.94,-0.52]** | **-484.52** | **-6.92** | **1.1059e-11** | **3.5810e-11** |
| **L inferior temporal** | **-0.902** | **0.108** | **[-1.11,-0.69]** | **-390.86** | **-8.60** | **6.5763e-17** | **8.9438e-16** |
| **R inferior temporal** | **-0.919** | **0.108** | **[-1.13,-0.71]** | **-350.55** | **-8.77** | **1.7820e-17** | **4.0392e-16** |
| **L isthmus cingulate** | **-0.604** | **0.106** | **[-0.81,-0.4]** | **-86.07** | **-5.76** | **1.3597e-08** | **2.3116e-08** |
| **R isthmus cingulate** | **-0.548** | **0.106** | **[-0.76,-0.34]** | **-68.35** | **-5.23** | **2.3512e-07** | **3.7182e-07** |
| **L lateral occipital** | **-0.613** | **0.106** | **[-0.82,-0.4]** | **-344.43** | **-5.84** | **8.3563e-09** | **1.4953e-08** |
| **R lateral occipital** | **-0.747** | **0.107** | **[-0.96,-0.54]** | **-425.79** | **-7.13** | **2.9215e-12** | **1.1686e-11** |
| **L lateral orbitofrontal** | **-0.714** | **0.107** | **[-0.92,-0.51]** | **-202.25** | **-6.81** | **2.2880e-11** | **7.0721e-11** |
| **R lateral orbitofrontal** | **-0.697** | **0.107** | **[-0.91,-0.49]** | **-223.06** | **-6.65** | **6.5200e-11** | **1.9276e-10** |
| **L lingual** | **-0.629** | **0.106** | **[-0.84,-0.42]** | **-236.96** | **-6.00** | **3.4445e-09** | **7.0977e-09** |
| **R lingual** | **-0.543** | **0.106** | **[-0.75,-0.34]** | **-215.91** | **-5.18** | **3.0394e-07** | **4.6973e-07** |
| **L medial orbitofrontal** | **-0.829** | **0.108** | **[-1.04,-0.62]** | **-164.93** | **-7.90** | **1.2734e-14** | **1.0824e-13** |
| **R medial orbitofrontal** | **-0.651** | **0.107** | **[-0.86,-0.44]** | **-130.75** | **-6.21** | **9.7128e-10** | **2.2016e-09** |
| **L middle temporal** | **-0.930** | **0.108** | **[-1.14,-0.72]** | **-349.07** | **-8.87** | **7.7755e-18** | **2.6438e-16** |
| **R middle temporal** | **-0.907** | **0.108** | **[-1.12,-0.7]** | **-362.47** | **-8.65** | **4.4270e-17** | **7.5259e-16** |
| **L parahippocampal** | **-0.418** | **0.106** | **[-0.63,-0.21]** | **-29.20** | **-3.99** | **7.5162e-05** | **8.2435e-05** |
| **R parahippocampal** | **-0.461** | **0.106** | **[-0.67,-0.25]** | **-34.00** | **-4.39** | **1.3062e-05** | **1.5861e-05** |
| **L paracentral** | **-0.605** | **0.106** | **[-0.81,-0.4]** | **-92.97** | **-5.77** | **1.2482e-08** | **2.1763e-08** |
| **R paracentral** | **-0.686** | **0.107** | **[-0.9,-0.48]** | **-114.68** | **-6.54** | **1.2577e-10** | **3.2735e-10** |
| **L pars opercularis** | **-0.353** | **0.105** | **[-0.56,-0.15]** | **-86.67** | **-3.37** | **8.0785e-04** | **8.1991e-04** |
| **R pars opercularis** | **-0.534** | **0.106** | **[-0.74,-0.33]** | **-101.96** | **-5.09** | **4.6615e-07** | **7.0440e-07** |
| **L pars orbitalis** | **-0.762** | **0.107** | **[-0.97,-0.55]** | **-66.58** | **-7.27** | **1.1295e-12** | **5.4861e-12** |
| **R pars orbitalis** | **-0.591** | **0.106** | **[-0.8,-0.38]** | **-62.55** | **-5.64** | **2.5947e-08** | **4.3034e-08** |
| **L pars triangularis** | **-0.393** | **0.106** | **[-0.6,-0.19]** | **-74.24** | **-3.75** | **1.9609e-04** | **2.0515e-04** |
| **R pars triangularis** | **-0.463** | **0.106** | **[-0.67,-0.26]** | **-101.61** | **-4.41** | **1.1993e-05** | **1.4827e-05** |
| **L pericalcarine** | **-0.460** | **0.106** | **[-0.67,-0.25]** | **-113.05** | **-4.39** | **1.3436e-05** | **1.6029e-05** |
| **R pericalcarine** | **-0.411** | **0.106** | **[-0.62,-0.2]** | **-102.43** | **-3.92** | **9.7627e-05** | **1.0373e-04** |
| **L postcentral** | **-0.783** | **0.107** | **[-0.99,-0.57]** | **-327.53** | **-7.46** | **2.9022e-13** | **1.6446e-12** |
| **R postcentral** | **-0.778** | **0.107** | **[-0.99,-0.57]** | **-309.34** | **-7.42** | **4.0204e-13** | **2.1030e-12** |
| **L posterior cingulate** | **-0.642** | **0.107** | **[-0.85,-0.43]** | **-100.27** | **-6.13** | **1.6078e-09** | **3.4165e-09** |
| **R posterior cingulate** | **-0.686** | **0.107** | **[-0.9,-0.48]** | **-106.79** | **-6.54** | **1.2998e-10** | **3.2735e-10** |
| **L precentral** | **-0.739** | **0.107** | **[-0.95,-0.53]** | **-352.05** | **-7.05** | **4.9562e-12** | **1.8724e-11** |
| **R precentral** | **-0.807** | **0.107** | **[-1.02,-0.6]** | **-345.41** | **-7.70** | **5.4592e-14** | **3.7123e-13** |
| **L precuneus** | **-0.833** | **0.108** | **[-1.04,-0.62]** | **-345.17** | **-7.95** | **9.2665e-15** | **9.0017e-14** |
| **R precuneus** | **-0.866** | **0.108** | **[-1.08,-0.66]** | **-374.23** | **-8.26** | **8.9995e-16** | **1.0199e-14** |
| **L rostral anterior cingulate** | **-0.625** | **0.106** | **[-0.83,-0.42]** | **-96.82** | **-5.96** | **4.3127e-09** | **8.6254e-09** |
| **R rostral anterior cingulate** | **-0.756** | **0.107** | **[-0.97,-0.55]** | **-92.20** | **-7.21** | **1.6764e-12** | **7.1246e-12** |
| **L rostral middle frontal** | **-0.727** | **0.107** | **[-0.94,-0.52]** | **-460.11** | **-6.93** | **1.0462e-11** | **3.5569e-11** |
| **R rostral middle frontal** | **-0.680** | **0.107** | **[-0.89,-0.47]** | **-470.59** | **-6.48** | **1.8550e-10** | **4.5050e-10** |
| **L superior frontal** | **-0.732** | **0.107** | **[-0.94,-0.52]** | **-556.23** | **-6.98** | **7.7326e-12** | **2.7675e-11** |
| **R superior frontal** | **-0.818** | **0.107** | **[-1.03,-0.61]** | **-614.07** | **-7.80** | **2.6235e-14** | **1.9822e-13** |
| **L superior parietal** | **-0.758** | **0.107** | **[-0.97,-0.55]** | **-471.71** | **-7.23** | **1.4385e-12** | **6.5214e-12** |
| **R superior parietal** | **-0.692** | **0.107** | **[-0.9,-0.48]** | **-399.07** | **-6.60** | **8.8312e-11** | **2.5022e-10** |
| **L superior temporal** | **-0.644** | **0.107** | **[-0.85,-0.44]** | **-269.43** | **-6.14** | **1.4409e-09** | **3.1607e-09** |
| **R superior temporal** | **-0.666** | **0.107** | **[-0.88,-0.46]** | **-242.47** | **-6.36** | **4.0480e-10** | **9.4918e-10** |
| **L supramarginal** | **-0.614** | **0.106** | **[-0.82,-0.41]** | **-370.79** | **-5.86** | **7.5770e-09** | **1.3925e-08** |
| **R supramarginal** | **-0.533** | **0.106** | **[-0.74,-0.33]** | **-247.22** | **-5.08** | **4.9108e-07** | **7.2594e-07** |
| **L frontal pole** | **-0.412** | **0.106** | **[-0.62,-0.2]** | **-13.95** | **-3.92** | **9.6560e-05** | **1.0373e-04** |
| **R frontal pole** | **-0.469** | **0.106** | **[-0.68,-0.26]** | **-20.29** | **-4.48** | **9.0568e-06** | **1.1405e-05** |
| **L temporal pole** | **-0.329** | **0.105** | **[-0.54,-0.12]** | **-21.98** | **-3.13** | **1.8021e-03** | **1.8021e-03** |
| **R temporal pole** | **-0.376** | **0.105** | **[-0.58,-0.17]** | **-25.56** | **-3.58** | **3.6960e-04** | **3.8080e-04** |
| **L transverse temporal** | **-0.501** | **0.106** | **[-0.71,-0.29]** | **-31.24** | **-4.78** | **2.1747e-06** | **3.0179e-06** |
| **R transverse temporal** | **-0.426** | **0.106** | **[-0.63,-0.22]** | **-18.65** | **-4.06** | **5.4434e-05** | **6.2738e-05** |
| **L insula** | **-0.452** | **0.106** | **[-0.66,-0.24]** | **-123.04** | **-4.31** | **1.9268e-05** | **2.2590e-05** |
| **R insula** | **-0.619** | **0.106** | **[-0.83,-0.41]** | **-135.00** | **-5.90** | **5.8888e-09** | **1.1441e-08** |

**Note.** General linear models per region controlling for age, sex, scan version, and T1w quality (MRIQC efc). FDR corrected using Benjamini-Hochberg procedure across all SA regions. Regions with *p*_FDR_<0.05 are depicted in bold. S2 = subtype 2, HC = healthy control, SE = standard error, L = left, R = right.

**Table S10a.** Subcortical volume comparisons among data-driven subtype 1 and healthy controls.

| Region | Cohen’s *d*  (S1 v. HC) | SE | 95% CI | Estimate | *t* value | *p*-value | FDR  *p*-value |
| --- | --- | --- | --- | --- | --- | --- | --- |
| L thalamus | 0.003 | 0.094 | [-0.18,0.19] | 1.64 | 0.03 | 0.97 | 0.98 |
| R thalamus | -0.02 | 0.094 | [-0.21,0.16] | -10.53 | -0.22 | 0.82 | 0.96 |
| **L caudate** | **0.49** | **0.095** | **[0.3,0.67]** | **186.63** | **5.17** | **3.12e-07** | **4.43e-06** |
| **R caudate** | **0.44** | **0.095** | **[0.25,0.63]** | **172.60** | **4.69** | **3.42e-06** | **2.39e-05** |
| **L putamen** | **0.24** | **0.094** | **[0.05,0.43]** | **141.29** | **2.55** | **0.01** | **0.03** |
| **R putamen** | **0.4** | **0.095** | **[0.21,0.58]** | **175.98** | **4.24** | **2.53e-05** | **1.18e-04** |
| **L pallidum** | **0.25** | **0.094** | **[0.06,0.43]** | **51.68** | **2.61** | **0.01** | **0.03** |
| **R pallidum** | **0.25** | **0.094** | **[0.06,0.43]** | **45.91** | **2.62** | **0.01** | **0.03** |
| L hippocampus | -0.002 | 0.094 | [-0.19,0.18] | -0.76 | -0.03 | 0.98 | 0.98 |
| R hippocampus | 0.06 | 0.094 | [-0.13,0.24] | 17.97 | 0.61 | 0.54 | 0.82 |
| L amygdala | 0.13 | 0.094 | [-0.06,0.31] | 24.95 | 1.36 | 0.17 | 0.31 |
| R amygdala | -0.027 | 0.094 | [-0.21,0.16] | -5.49 | -0.29 | 0.78 | 0.96 |
| L accumbens | 0.05 | 0.094 | [-0.13,0.24] | 5.16 | 0.55 | 0.59 | 0.82 |
| R accumbens | 0.15 | 0.094 | [-0.04,0.33] | 12.87 | 1.59 | 0.11 | 0.23 |

**Note.** General linear models per region controlling for age, sex, scan version, T1w quality (MRIQC efc), and intracranial volume. **p*<0.05, ***p*<0.01, ****p*<0.001. FDR corrected using Benjamini-Hochberg procedure across all SV regions. Regions with *p*_FDR_<0.05 are depicted in bold. S1 = subtype 1, HC = healthy control, SE = standard error, L = left, R = right.

**Table S10b.** Subcortical volume comparisons among data-driven subtype 2 and healthy controls.

| Region | Cohen’s *d*  (S2 v. HC) | SE | 95% CI | Estimate | *t* value | *p*-value | FDR  *p*-value |
| --- | --- | --- | --- | --- | --- | --- | --- |
| **L thalamus** | **-0.79** | **0.112** | **[-1.01,-0.57]** | **-433.46** | **-7.22** | **1.51e-12** | **4.21e-12** |
| **R thalamus** | **-0.77** | **0.112** | **[-0.99,-0.55]** | **-387.45** | **-7.02** | **5.98e-12** | **1.40e-11** |
| **L caudate** | **-0.33** | **0.110** | **[-0.55,-0.11]** | **-126.30** | **-3.00** | **0.003** | **0.004** |
| **R caudate** | **-0.34** | **0.110** | **[-0.55,-0.12]** | **-131.60** | **-3.06** | **0.002** | **0.003** |
| **L putamen** | **-0.33** | **0.110** | **[-0.55,-0.11]** | **-194.80** | **-3.01** | **0.003** | **0.004** |
| R putamen | -0.17 | 0.110 | [-0.38,0.05] | -72.98 | -1.51 | 0.13 | 0.13 |
| L pallidum | -0.19 | 0.110 | [-0.4,0.03] | -39.14 | -1.70 | 0.09 | 0.10 |
| R pallidum | -0.21 | 0.110 | [-0.43,0.01] | -39.17 | -1.91 | 0.06 | 0.07 |
| **L hippocampus** | **-0.93** | **0.113** | **[-1.15,-0.7]** | **-290.32** | **-8.44** | **2.34e-16** | **1.61e-15** |
| **R hippocampus** | **-0.93** | **0.113** | **[-1.15,-0.71]** | **-291.35** | **-8.47** | **1.82e-16** | **1.61e-15** |
| **L amygdala** | **-0.89** | **0.113** | **[-1.11,-0.67]** | **-173.39** | **-8.08** | **3.41e-15** | **1.59e-14** |
| **R amygdala** | **-0.83** | **0.112** | **[-1.05,-0.61]** | **-170.29** | **-7.57** | **1.41e-13** | **4.94e-13** |
| **L accumbens** | **-0.53** | **0.111** | **[-0.75,-0.32]** | **-53.74** | **-4.86** | **1.50e-06** | **2.62e-06** |
| **R accumbens** | **-0.55** | **0.111** | **[-0.77,-0.33]** | **-47.61** | **-5.03** | **6.46e-07** | **1.29e-06** |

**Note.** General linear models per region controlling for age, sex, scan version, T1w quality (MRIQC efc), and intracranial volume. **p*<0.05, ***p*<0.01, ****p*<0.001. FDR corrected using Benjamini-Hochberg procedure across all SV regions. Regions with *p*_FDR_<0.05 are depicted in bold. S2 = subtype 2, HC = healthy control, SE = standard error, L = left, R = right.

REFERENCES

1.	First, M., Spitzer, R., Gibbon, M., Williams, J., *Structured clinical interview for DSM-IV-TR axis I disorders, research version, patient edition (SCID-I/P).* New York: Biometrics Research, 2002.

2.	Markello, R.D., et al., *Multimodal phenotypic axes of Parkinson's disease.* Npj Parkinsons Disease, 2021. **7**(1).

3.	Gonul, A.S., C. Candemir, and P. Thompson, *Subtyping schizophrenia via machine learning by using structural neuroimaging.* Transl Psychiatry, 2025. **15**(1): p. 472.

4.	Jorstad, N.L., et al., *Transcriptomic cytoarchitecture reveals principles of human neocortex organization.* Science, 2023. **382**(6667): p. eadf6812.

5.	Hawrylycz, M.J., et al., *An anatomically comprehensive atlas of the adult human brain transcriptome.* Nature, 2012. **489**(7416): p. 391-399.
